# Regulation of the urea cycle by CPS1 *O*-GlcNAcylation in response to dietary restriction and aging

**DOI:** 10.1093/jmcb/mjac016

**Published:** 2022-03-14

**Authors:** Jing Wu, Jiayu Liu, Kalina Lapenta, Reina Desrouleaux, Min-Dian Li, Xiaoyong Yang

**Affiliations:** Department of Comparative Medicine, Department of Cellular and Molecular Physiology, and Yale Center for Molecular and Systems Metabolism, Yale University School of Medicine, New Haven, CT 06520, USA; School of Life Science and Technology, Xi'an Jiaotong University, Xi'an 710049, China; Department of Comparative Medicine, Department of Cellular and Molecular Physiology, and Yale Center for Molecular and Systems Metabolism, Yale University School of Medicine, New Haven, CT 06520, USA; Department of Comparative Medicine, Department of Cellular and Molecular Physiology, and Yale Center for Molecular and Systems Metabolism, Yale University School of Medicine, New Haven, CT 06520, USA; Department of Comparative Medicine, Department of Cellular and Molecular Physiology, and Yale Center for Molecular and Systems Metabolism, Yale University School of Medicine, New Haven, CT 06520, USA; Department of Comparative Medicine, Department of Cellular and Molecular Physiology, and Yale Center for Molecular and Systems Metabolism, Yale University School of Medicine, New Haven, CT 06520, USA; Department of Comparative Medicine, Department of Cellular and Molecular Physiology, and Yale Center for Molecular and Systems Metabolism, Yale University School of Medicine, New Haven, CT 06520, USA

**Keywords:** ageing, *O*-GlcNAcylation, urea cycle, carbamoyl phosphate synthetase 1, calorie restriction, posttranslational modification, dietary restriction

## Abstract

*O*-linked *N*-acetyl-glucosamine glycosylation (*O*-GlcNAcylation) of intracellular proteins is a dynamic process broadly implicated in age-related disease, yet it remains uncharacterized whether and how *O*-GlcNAcylation contributes to the natural aging process. *O*-GlcNAc transferase (OGT) and the opposing enzyme *O*-GlcNAcase (OGA) control this nutrient-sensing protein modification in cells. Here, we show that global *O*-GlcNAc levels are increased in multiple tissues of aged mice. In aged liver, carbamoyl phosphate synthetase 1 (CPS1) is among the most heavily *O*-GlcNAcylated proteins. CPS1 *O*-GlcNAcylation is reversed by calorie restriction and is sensitive to genetic and pharmacological manipulations of the *O*-GlcNAc pathway. High glucose stimulates CPS1 *O*-GlcNAcylation and inhibits CPS1 activity. Liver-specific deletion of OGT potentiates CPS1 activity and renders CPS1 irresponsive to further stimulation by a prolonged fasting. Our results identify CPS1 *O*-GlcNAcylation as a key nutrient-sensing regulatory step in the urea cycle during aging and dietary restriction, implying a role for mitochondrial *O*-GlcNAcylation in nutritional regulation of longevity.

## Introduction

Nutrient flux through the hexosamine biosynthetic pathway (HBP) leads to dynamic and reversible protein modification by single *O*-linked β-*N*-acetylglucosamine (*O*-GlcNAc) moieties at serine or threonine residues of nuclear, cytoplasmic, and mitochondrial proteins, termed *O*-GlcNAcylation (Yang and Qian, 2017; Chatham et al., 2021; Ma et al., 2021). In mammals, *O-*GlcNAc is added and removed by the enzymes *O-*GlcNAc transferase (OGT) and *O*-GlcNAcase (OGA), respectively (Alteen et al., 2021; Stephen et al., 2021). *O*-GlcNAcylation is a nutrient sensor and can directly modulate metabolic pathways via modification of metabolic enzymes (Yang and Qian, 2017; Hart, 2019). Phosphofructokinase 1 activity is decreased by *O*-GlcNAcylation at the site Ser529, which redirects glucose flux from the glycolytic pathway to the oxidative pentose phosphate pathway (Yi et al., 2012). Ablation of the *ogt* allele in *Caenorhabditis elegans* impairs metabolism and longevity (Lee et al., 2010; Rahman et al., 2010). Aberrant *O*-GlcNAcylation has been implicated in a spectrum of age-related diseases in humans and rats, including obesity, diabetes, cancer, cardiovascular disease, and Alzheimer's disease (Rahman et al., 2010; Marotta et al., 2012; Jahangir et al., 2014; Jozwiak et al., 2014; Yuzwa et al., 2014; Li et al., 2018). It has been reported that the levels of protein *O*-GlcNAcylation were increased in brain and muscle tissues in aged Brown–Norway rats (Fulop et al., 2008). Neuronal OGT can rejuvenate cognitive decline in aged mice (Wheatley et al., 2019). Although these diseases are tightly associated with aging, it remains unclarified whether, and if so how, *O*-GlcNAcylation is broadly involved in the etiology of aging.

As a hallmark and accelerator of aging, mitochondrial dysfunction manifests as metabolic perturbations in the tricarboxylic acid (TCA) cycle, fatty acid oxidation, oxidative phosphorylation (OXPHOS), ketogenesis, and amino acid catabolism (Bratic and Trifunovic, 2010). Mitochondrial metabolism plays an essential role in the life-extending actions of dietary restriction, including calorie restriction (CR) and prolonged, periodic fasting (Wilson et al., 2021; Green et al., 2022). Deleting the LAT1 gene, encoding dihydrolipoamide acetyltransferase, abolishes a chronological lifespan extension induced by CR (Easlon et al., 2007). CR reduces oxidative stress and upregulates mitochondrial biogenesis via peroxisome proliferator-activated receptor-γ coactivator-1α activation (Lopez-Lluch et al., 2006). Nutrient-sensing mechanisms involved in CR include insulin-like growth factor 1 signaling, adenosine 5′-monophosphate-activated protein kinase, and mammalian target of rapamycin complex (Wilson et al., 2021). It is unknown whether there exist additional nutrient sensors.

Protein posttranslational modifications (PTMs) are the key regulatory mechanisms that control mitochondrial metabolism (Hofer and Wenz, 2014). For example, protein acetylation has been known as a reversible mechanism linking metabolic state and mitochondrial function (Ryu et al., 2014). Early studies reported that levels of mitochondrial *O*-GlcNAcylation were very low. However, there is increasing evidence that *O*-GlcNAcylation is pervasive among mitochondrial proteins and potentially essential for mitochondrial functions (Ruan et al., 2013; Tan et al., 2014a; Jozwiak et al., 2021). Some *O*-GlcNAcylated mitochondrial proteins have been identified in rat liver, the majority of which are involved in the urea cycle, TCA cycle, and lipid metabolism (Cao et al., 2013). Overexpressing OGT/OGA can inhibit cellular respiration and glycolysis indicating profound effects on energy and metabolite production (Jozwiak et al., 2014). Increased *O*-GlcNAcylation of the respiratory chain complexes I, III, and IV impairs mitochondrial energy production in cardiac myocytes (Hu et al., 2009). Furthermore, low-running-capacity rats exhibit increased *O*-GlcNAcylation of cardiac proteins, including mitochondrial complex I and complex IV, VDAC, and sarco/endoplasmic reticulum Ca^2+^-ATPase (Johnsen et al., 2013). However, it is unknown how *O*-GlcNAcylation regulates mitochondrional function other than OXPHOS.

To elucidate whether and how *O*-GlcNAc signaling contributes to the aging process, we profiled the levels of *O*-GlcNAc in four metabolic tissues of young and aged mice, and identified 10 *O*-GlcNAc-targeted mitochondrial proteins in aged mouse liver through mass spectrometry (MS). Carbamoyl phosphate synthetase 1 (CPS1), the first and rate-limiting enzyme in the urea cycle, is the most abundant *O*-GlcNAcylated protein in the list. We found that CR reduces the global *O*-GlcNAc levels as well as CPS1 *O*-GlcNAcylation in the liver. Next, we focused on site mapping and regulation of CPS1. High glucose and inhibition of OGA increase the levels of CPS1 *O*-GlcNAcylation and repress CPS1 activity. Overexpression of OGT increases CPS1 *O*-GlcNAcylation, while depletion of endogenous OGT or induction of OGA expression in cells eliminates CPS1 *O*-GlcNAcylation. OGT and OGA control the dynamic cycling of *O*-GlcNAc by direct interaction with CPS1. We validated CPS1 *O*-GlcNAcylation in mouse liver and showed that liver-specific OGT knockout enhances CPS1 activity and perturbs the stimulatory effects of 48 h fasting on ammonia detoxification. These findings demonstrate the alteration of protein *O*-GlcNAcylation in a broad range of tissues in the aging process and reveal mitochondrial *O*-GlcNAcylation as a regulatory mechanism by which dietary restriction regulates the urea cycle during aging.

## Results

### Identification of potential O-GlcNAc modification targets in aged mice

**Figure 1:**
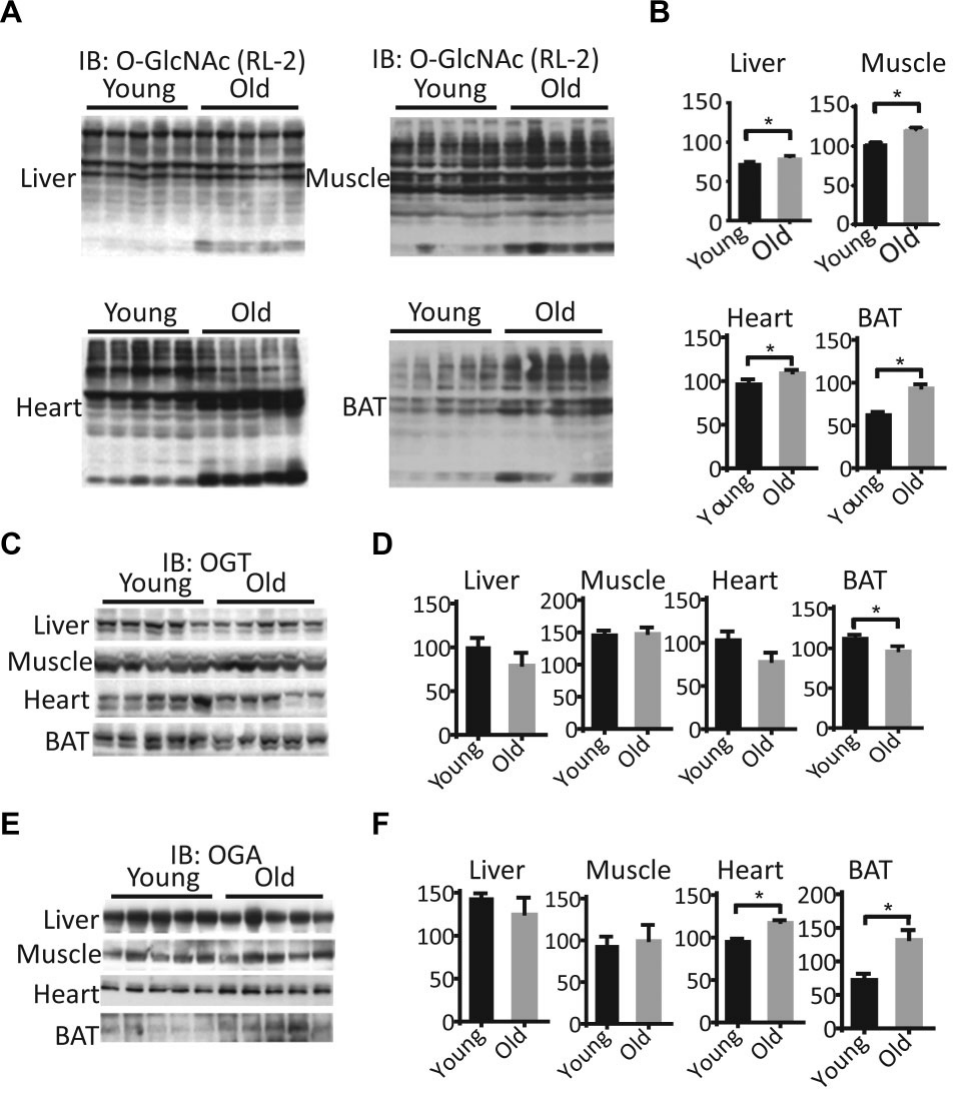
Alterations of tissue *O*-GlcNAcylation in aged mice. The protein levels of global *O*-GlcNAc (**A** and **B**), OGT (**C** and **D**), and OGA (**E** and **F**) were detected in the liver, heart, muscle, and BAT from mice at 4 (young) and 24 (old) months old. (**A, C**, and **E**) Representative western blots. (**B, D**, and **F**) Mean area densities of *O*-GlcNAc (**B**), OGT (**D**), and OGA (**F**). *P < 0.05, old vs. young.

To explore the role of dynamic *O*-GlcNAc cycling in the aging process, we obtained tissues from young (4-month-old) and old (24-month-old) mice, and analyzed *O*-GlcNAc signaling in the liver, heart, skeletal muscle, and brown adipose tissue (BAT). Although no significant change in OGT or OGA expression was observed, global increases in *O*-GlcNAc levels in the tissues from old mice suggest that the activities of OGT and OGA are possibly modulated in aged mice (Figure 1A–F).

**Figure 2:**
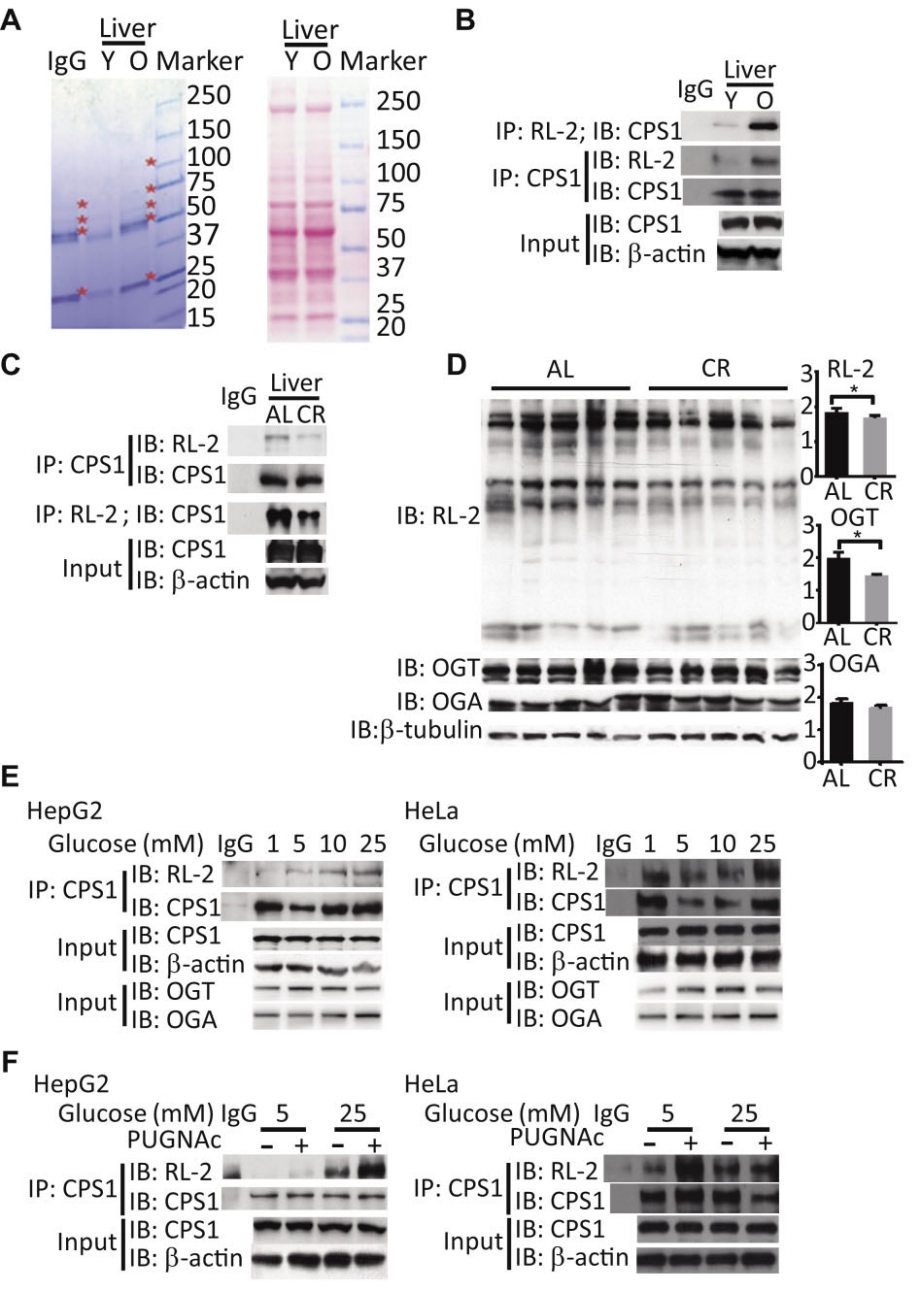
CPS1 is a target of *O*-GlcNAcylation in aged liver. (**A**) Coomassie blue staining identifies hyper-*O*-GlcNAcylated proteins precipitated by *O*-GlcNAc antibody from liver homogenates of young (Y) and old (O) mice (left panel). Asterisks indicate protein bands with differential intensity. Ponceau S staining shows the equal input (right panel). (**B**) CPS1 *O*-GlcNAc was detected by reciprocal IP in liver homogenates from young and old mice. (**C**) CR downregulated CPS1 *O*-GlcNAcylation. AL, ad libitum. (**D**) CR decreased global levels of *O*-GlcNAc in the liver. (**E**) HepG2 cells and HeLa cells were cultured with different concentrations of glucose overnight. The levels of CPS1 *O*-GlcNAc were elevated by high glucose. (**F**) HepG2 cells were cultured with low (5 mM) and high (25 mM) glucose, in the presence or absence of PUGNAc overnight. Excessive CPS1 *O*-GlcNAcylation was induced by high glucose and PUGNAc. CPS1 *O*-GlcNAcylation was increased by the OGA inhibitor PUGNAc.

To delineate the pathways in which *O*-GlcNAc modification contributes to aging, we identified potential *O*-GlcNAcylated proteins from the liver using immunoprecipitation (IP) and MS-based proteomics. Liver proteins (*n* = 5) were pulled down by an *O*-GlcNAc antibody (RL-2) and separated by sodium dodecyl sulphate–polyacrylamide gel electrophoresis. Coomassie blue staining showed that several protein bands were significantly more intense in the livers of old mice (Figure 2A). By using MS, we identified these *O*-GlcNAc targets enriched in the aged liver, which are involved in ATP generation (ATP5a1 and ATP5b), the urea cycle (CPS1), detoxification (GSTP1 and GSTMu1), the chaperonin family (HSPD1, HSPA5, HSPA9, and HSPA8), and cytokinesis (MYH9) (Table 1). The identified mitochondrial enzymes indicate that *O*-GlcNAcylation may be an important regulator of mitochondrial metabolism and bioenergetics during aging.

**Table 1:** Identification of potential *O*-GlcNAc targets in aged liver.

ID	Score	Expectation	Protein ID	Protein name	Molecular weight	%Coverage
L-O-1	665	4.80E−63	GSTP1_MOUISE	Glutathione *S*-transferase P 1 OS=*Mus musculus* GN=Gstp1 PE=1 SV=2	23594	53.3
L-O-1	581	1.20E−54	GSTM1_MOUISE	Glutathione S-transferase Mu 1 OS=*Mus musculus* GN=Gstm1 PE=1 SV=2	25953	60.6
L-O-2	721	1.20E−68	ATPA_MOUISE	ATP synthase subunit alpha, mitochondrial OS=*Mus musculus* GN=Atp5a1 PE=1 SV=1	59716	32.5
L-O-2	695	5.10E−66	ATPB_MOUISE	ATP synthase subunit beta, mitochondrial OS=*Mus musculus* GN=Atp5b PE=1 SV=2	56265	35.5
L-O-3	535	4.80E−50	CH60_MOUISE	60 kDa heat shock protein, mitochondrial OS=*Mus musculus* GN=Hspd1 PE=1 SV=1	60917	32.5
L-O-4	964	7.10E−93	GPR78_MOUISE	78 kDa glucose-regulated protein OS=*Mus musculus* GN=Hspa5 PE=1 SV=3	72377	40.8
L-O-4	857	3.10E−82	GPR75_MOUISE	Stress-70 protein, mitochondrial OS=*Mus musculus* GN=Hspa9 PE=1 SV=2	73483	31.7
L-O-4	754	7.20E−72	HSP7C_MOUISE	Heat shock cognate 71 kDa protein OS=*Mus musculus* GN=Hspa8 PE=1 SV=1	70827	40.1
L-O-5	2917	0	CPSM_MOUISE	Carbamoyl phosphate synthase (ammonia), mitochondrial OS=*Mus musculus* GN=Cps1 PE=1 SV=2	164514	46.2
L-O-6	3754	0	MYH9_MOUISE	Myosin-9 OS=*Mus musculus* GN=Myh9 PE=1 SV=4	226232	45.2

Among the *O*-GlcNAcylated proteins in the list, CPS1 accounts for 20% of the protein mass in the mitochondrial matrix and gates the entry into the urea cycle (Crouser et al., 2006). To confirm the *O*-GlcNAcylation of CPS1, we carried out reciprocal IP for liver extracts from young and old mice with the CPS1 antibody and blotted with the RL-2 antibody. The results showed that the level of CPS1 *O*-GlcNAcylation was much higher in old mice than in young mice, but the protein levels were not changed significantly (Figure 2B). We also observed that mice subjected to 40% CR for 6 months exhibited decreased levels of global *O*-GlcNAc, CPS1 *O*-GlcNAcylation, and OGT in the liver without altering the level of OGA (Figure 2C and D). In general, the magnitude of *O*-GlcNAcylation of intracellular proteins correlates with extracellular glucose levels (Liu et al., 2000). We examined the levels of CPS1 *O*-GlcNAcylation in response to different concentrations of glucose. The result showed that high glucose (25 mM) greatly elevated CPS1 *O*-GlcNAcylation (Figure 2E). Levels of OGA protein increased in a dose-dependent manner with regard to glucose concentration, whereas levels of OGT protein exhibited a bell-shaped response to glucose concentration (Figure 2E). Chemical inhibition of OGA activity by *O*-(2-acetamido-2-deoxy-D-glucopyranoslidene)amino-*N*-phenylcarbamate (PUGNAc) overnight further increased the levels of CPS1 *O*-GlcNAcylation (Figure 2F). These results showed that CPS1 is an *O-*GlcNAc-targeted protein that is sensitive to glucose availability.

### OGT and OGA regulate CPS1 O-GlcNAcylation

**Figure 3:**
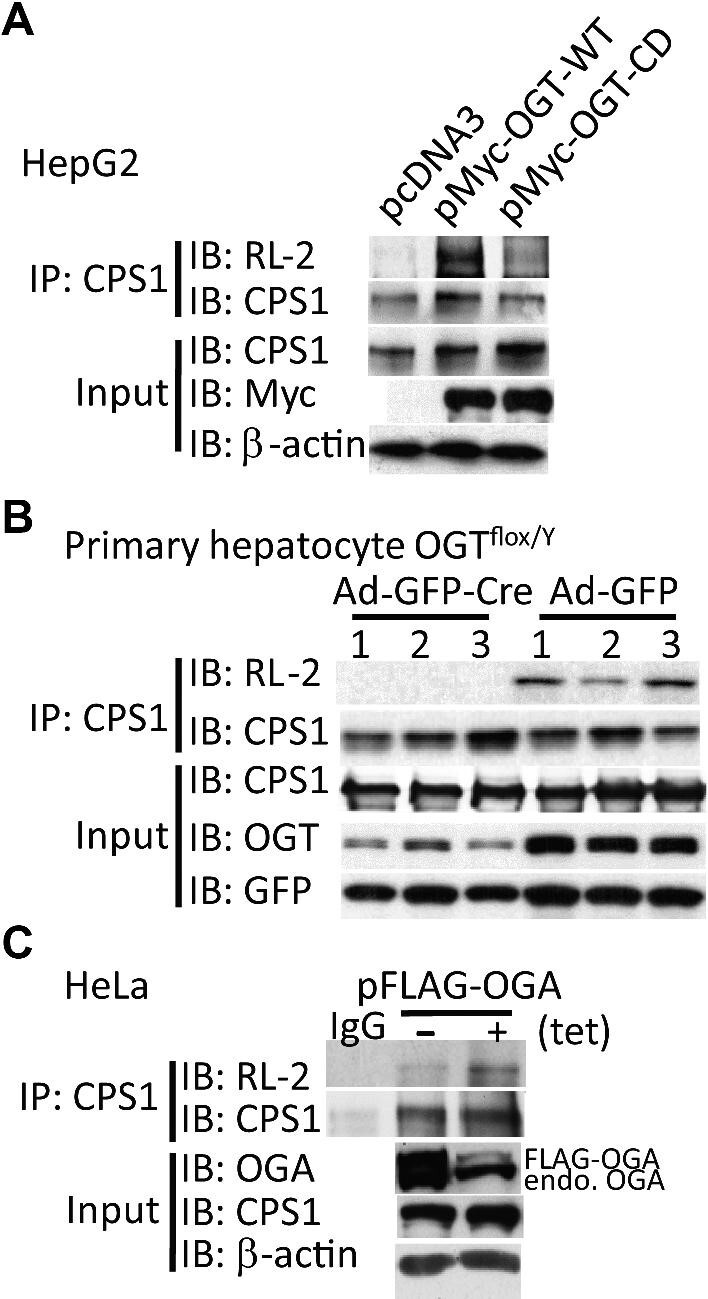
OGT and OGA regulate CPS1 *O*-GlcNAcylation. (**A**) HepG2 cells were transfected with plasmids expressing wild-type (pMyc-OGT-WT) or catalytically dead OGT (pMyc-OGT-CD). Overexpression of wild-type OGT increased the levels of CPS1 *O*-GlcNAc. (**B**) Primary hepatocytes isolated from OGT^flox/Y^ mice were infected with adenovirus encoding Cre for OGT knockout or with Ad-GFP as control. OGT knockout eliminated CPS1 *O*-GlcNAc in primary hepatocytes. (**C**) HeLa cells were stably transfected with pTRE2pur-3Flag/2Myc-OGA (WT) and pTet-Off plasmids. OGA expression was induced by removal of doxycycline. OGA overexpression decreased CPS1 *O*-GlcNAc.

In order to ascertain the effect of OGT on CPS1 *O*-GlcNAcylation, HepG2 cells were transfected with pCMV-Myc-OGT wild-type or pCMV-Myc-OGT catalytically dead plasmids (Chen et al., 2013). As shown in Figure 3A, CPS1 *O*-GlcNAcylation was significantly increased by OGT overexpression; however, the catalytically dead mutant of OGT only slightly upregulated CPS1 *O*-GlcNAcylation. The primary hepatocytes were isolated from the livers of OGT^flox/Y^ male mice and infected by adenoviruses encoding Cre, with adenovirus green fluorescent protein (Ad-GFP) as the control. In OGT^flox/Y^ mouse primary hepatocytes subjected to Cre-induced homologous recombination that eliminates OGT expression, the expression of CPS1 was unchanged, but *O*-GlcNAcylation of CPS1 was downregulated as demonstrated by IP analysis (Figure 3B). These results indicate that CPS1 is the substrate of OGT. To examine whether OGA catalyzes the removal of *O*-GlcNAc from CPS1, HeLa cells were stably transfected with pTRE2pur-3Flag/2Myc-OGA (WT) and pTet-Off plasmids. As shown in Figure 3C, OGA expression was induced by removal of doxycycline, which significantly decreased *O*-GlcNAcylation of CPS1.

### OGT and OGA interact with CPS1

**Figure 4:**
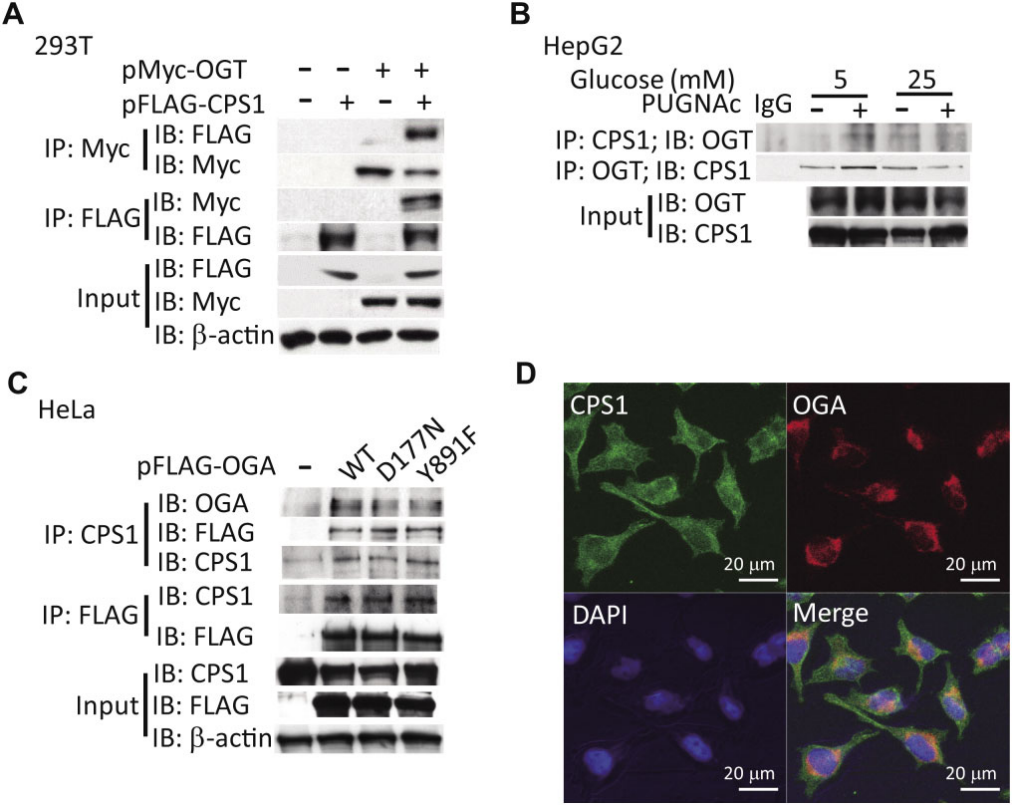
Physical interaction between OGT, OGA, and CPS1. (**A**) 293T cells were transfected with Myc-tagged OGT and/or FLAG-tagged CPS1 expression vectors, and co-IP was performed with anti-Myc and anti-FLAG antibody. (**B**) HepG2 cells were cultured with low and high concentrations of glucose for 16 h. IP was performed with anti-CPS1 and anti-OGT antibody. Endogenous CPS1 and OGT were reciprocally immunoprecipitated. (**C**) FLAG-tagged wild-type, D177N, or Y891F OGA was overexpressed in HeLa cells. Co-IP was performed with anti-CPS1 and anti-FLAG antibody. OGA was coimmunoprecipitated with CPS1. (**D**) HeLa cells were immunostained with anti-CPS1 and anti-OGA antibody. OGA and CPS1 were colocalized as determined by confocal microscopy.

To assess the physical interaction between OGT and CPS1, we coexpressed FLAG-tagged CPS1 and Myc-tagged OGT in HEK293T cells. The result showed that OGT was coimmunoprecipitated with CPS1, demonstrating that CPS1 can physically associate with OGT (Figure 4A). Direct protein–protein interactions between CPS1 and OGT were confirmed by reciprocal IP of endogenous CPS1 with endogenous OGT in HepG2 cells (Figure 4B). To test whether OGA physically interacts with CPS1, we overexpressed wild-type, D177N mutant, or Y891F mutant OGA with FLAG tagging in HeLa cells. The data showed that endogenous CPS1 was coimmunoprecipitated with wild-type OGA and OGA mutants (Figure 4C). D177N mutant OGA is *O*-GlcNAcase catalytically dead, and Y891F mutant OGA is histone acetyltransferase catalytically dead (Toleman et al., 2004; Singh et al., 2020). Our data indicated that the interaction between OGA and CPS1 is not affected by the point mutation on OGA Asp177 or Tyr891 residue. Previous report has confirmed the presence of OGA in rat cardiac mitochondria (Banerjee et al., 2015). Our result of immunostaining and confocal microscopy showed that OGA and CPS1 were colocalized in mammalian cells (Figure 4D).

### O-GlcNAc modification of CPS1 inhibits its enzymatic activity

**Figure 5:**
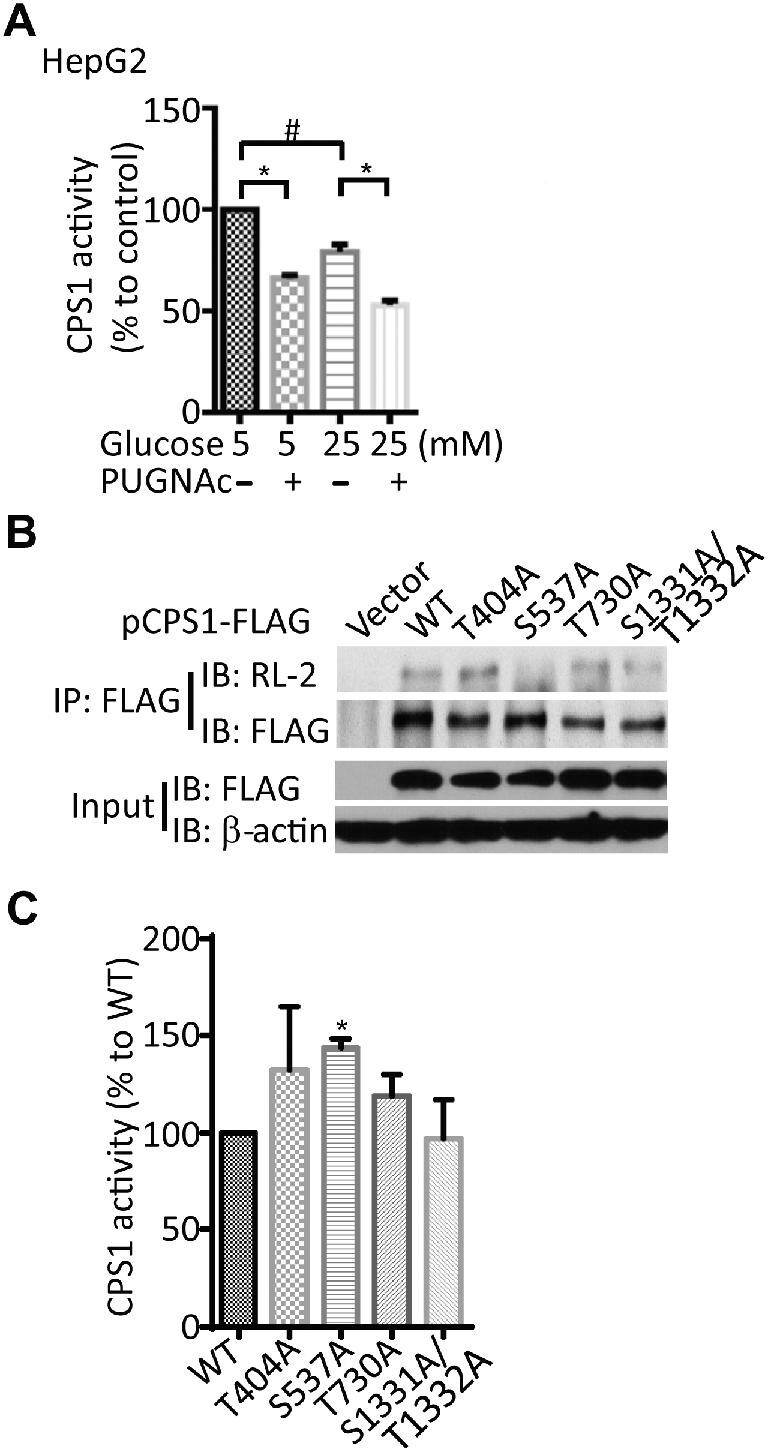
*O*-GlcNAcylation of CPS1 modulates its activity. (**A**) CPS1 activity was suppressed by high glucose and PUGNAc-induced *O*-GlcNAcylation. Data shown are mean ± SEM of three independent experiments. ^#^*P* < 0.05, low glucose vs. high glucose; *P < 0.05. (**B**) CPS1 alanine-scanning mutagenesis. 293T cells were transfected with empty vector or FLAG-tagged CPS1 plasmids in which Thr404, Ser537, Thr730, and Ser1331/Thr1332 sites were individually or doubly replaced with Ala. CPS1 was immunoprecipitated using anti-FLAG antibody, and *O*-GlcNAc of CPS1 was analyzed by IB with RL-2 antibody. (**C**) Mitochondria were isolated from 293T cells transfected with plasmids expressing wild-type CPS1 or CPS1 mutants, and CPS1 activity was assessed by OTC-coupled assay. Data are shown as mean ± SEM of three independent experiments. *P < 0.05, wild-type vs. mutant.

PTMs play a pivotal role in regulating enzymatic activity (Gao et al., 2014; Hofer and Wenz, 2014). It has been reported that CPS1 has multiple types of PTMs, such as acetylation, succinylation, and glutarylation, which inhibit CPS1 activity (Nakagawa et al., 2009; Du et al., 2011; Tan et al., 2014b). We observed that the activity of CPS1 in HepG2 cells was inhibited by high glucose and the treatment of PUGNAc (Figure 5A). Our data suggested that *O*-GlcNAcylation of CPS1 may be involved in the regulation of the urea cycle and amino acid catabolism.

### Verification of CPS1 O-GlcNAcylation sites

Three *O*-GlcNAcylation sites have been identified, i.e. Ser537, Ser1331, and Thr1332, but the function of these *O*-GlcNAc sites remains uncharacterized (Cao et al., 2013). To determine the contribution of these sites to overall *O*-GlcNAcylation of CPS1, we constructed FLAG-tagged CPS1 in which Thr404, Ser537, Thr730, and Ser1331/Thr1332 sites were individually or doubly replaced with alanine to abolish potential *O*-GlcNAcylation. Ectopically expressed CPS1 mutants were immunoprecipitated with anti-FLAG antibody, and *O*-GlcNAc was detected using the RL-2 antibody. The results showed that the mutation at Ser537 was the only one that considerably reduced CPS1 *O*-GlcNAcylation (Figure 5B). To further confirm the function of *O*-GlcNAc sites on CPS1, we assessed the activity of CPS1 mutants. After transfection of FLAG-tagged CPS1 mutants for 48 h, mitochondria were isolated and CPS1 activity was assessed by ornithine aminotransferase (OTC)-coupled assay. We observed that the S537A mutant of CPS1 showed a significantly higher activity than the wild-type CPS1 (Figure 5C).

### O-GlcNAc inhibits CPS1 and ammonia detoxification in mice

**Figure 6:**
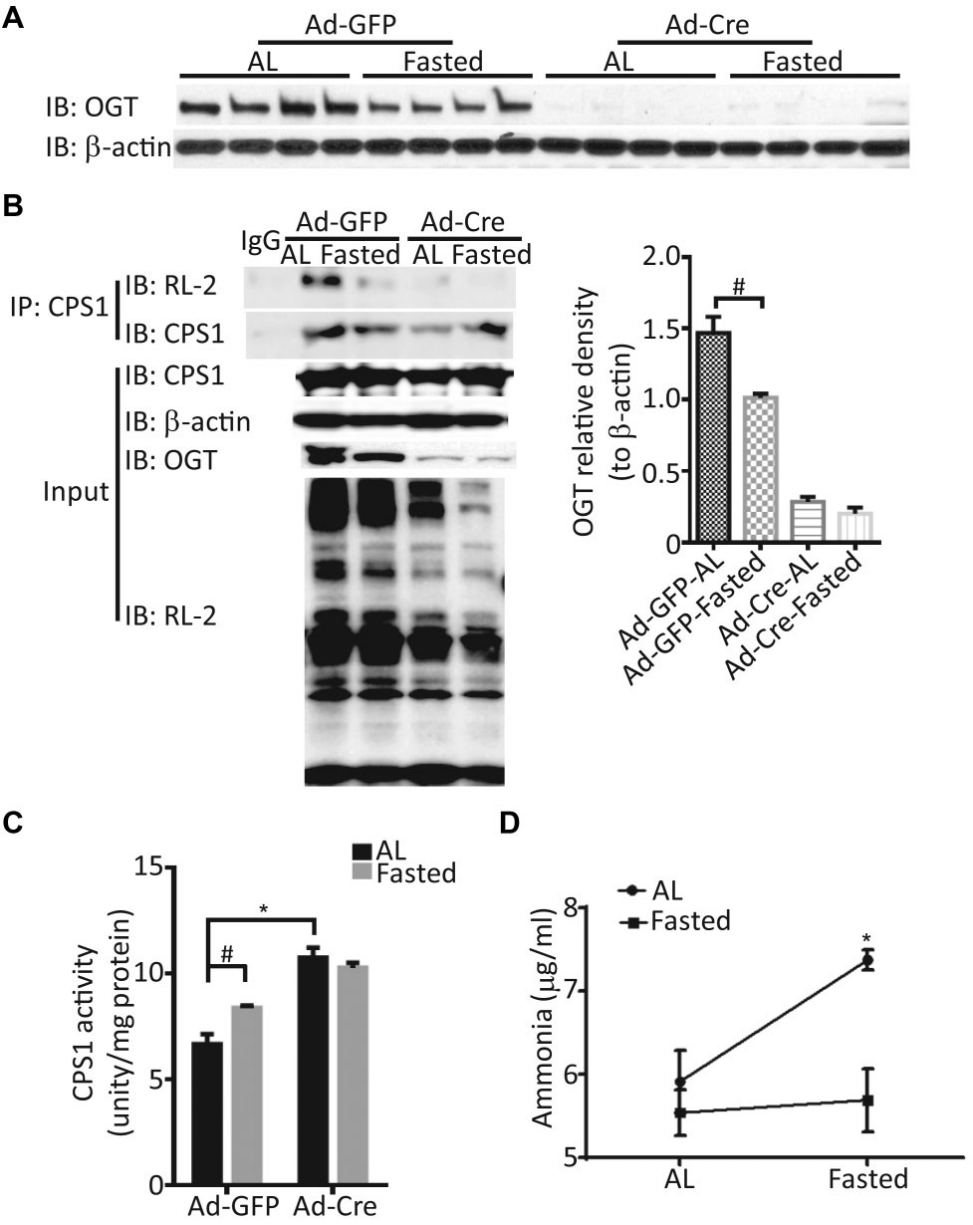
Prolonged fasting upregulates CPS1 activity through OGT downregulation *in vivo*. Liver-specific OGT knockout mice were generated by tail vein injection of the recombinant adenovirus expressing Cre recombinase (Ad-Cre) into OGT^floxed/Y^ mice, with Ad-GFP as control. After 4–7 days, mice were either fasted for 48 h or with normal chow (AL) as control. (**A**) OGT protein levels in the liver were decreased after 48 h fasting. Mean area densities of OGT are shown (lower graph). *n* = 4 for each group, ^#^*P* < 0.05. (**B**) The levels of CPS1 *O*-GlcNAcylation were analyzed by IP and western blotting using anti-CPS1 antibody and the RL-2 antibody. (**C**) CPS1 activity was measured. Error bars represent SEM (*n* = 4 for each group). Prolonged fasting induced CPS1 activity in wild-type. In OGT knockout mice, CPS1 activity was enhanced by lessened *O*-GlcNAcylation, but not further induced by prolonged fasting. (**D**) The levels of serum ammonia were measured by an Ammonia Colorimetric Assay Kit. Error bars represent SEM (*n* = 4 for each group). Prolonged fasting caused hyperammonia in wild-type but not OGT knockout mice.

To examine the physiological importance of OGT in mouse liver, we generated liver-specific OGT knockout mice by tail-vein injection of the recombinant adenovirus expressing Cre recombinase into OGT^floxed/Y^ mice as described (Ruan et al., 2017). We predicted that the deficiency of OGT might influence metabolic adaptation to prolonged fasting, which triggers amino acid catabolism. The data showed that OGT protein expression was decreased after 48 h fasting in wild-type mice (Figure 6A). CPS1 was immunoprecipitated from the mixture of liver homogenates and detected by the RL-2 antibody. The level of CPS1 *O*-GlcNAcylation was significantly reduced in response to 48 h fasting and was almost eliminated by OGT knockout (Figure 6B).

CPS1 is the key enzyme for ammonia detoxification. It has been observed that CR increases CPS1 enzyme activity by at least 2-fold in the mouse liver, which is considered to facilitate the turnover of extrahepatic proteins (Dhahbi et al., 2001). We observed that CPS1 activity was significantly elevated by 48 h fasting in wild-type mice; however, in hepatic OGT knockout mice, the increased CPS1 activity could not be further induced by fasting (Figure 6C). This is corroborated by the ammonia level in the blood of OGT-deficient mice during prolonged fasting (Figure 6D). In the fed condition, OGT knockout and wild-type mice showed comparable blood ammonia levels. After 48 h fasting, wild-type mice showed significantly elevated blood ammonia, but OGT knockout mice kept ammonia levels comparable to the fed control (Figure 6D). The results indicated that decreased *O*-GlcNAcylation upregulated CPS1 activity to keep the fluctuations of blood ammonia within the physiological range. Together, these findings suggest that dietary restriction facilitates protein catabolism and ammonia disposal in part through reducing *O*-GlcNAcylation of CPS1.

## Discussion

Protein *O*-GlcNAcylation is a nutrient sensor that is broadly studied in nuclear and cytoplasmic compartments, yet mitochondrial *O*-GlcNAcylation is less characterized (Zachara and Hart, 2004; Harwood and Hanover, 2014; Yang and Qian, 2017). Aberrant *O*-GlcNAcylation is associated with aging and age-related diseases (Rahman et al., 2010; Yuzwa et al., 2014). In this study, we identified a number of *O*-GlcNAcylated mitochondrial proteins in aged tissues through proteome profiling. These proteins play key roles in mitochondrial metabolism (i.e. CPS1), bioenergetics (i.e. ATP synthase), and stress response (i.e. glutathione S-transferase and heat shock proteins). Aging increased the level of CPS1 protein 8-fold in mouse adipose tissue (Plubell et al., 2017) and 1.5/2-fold in rat brain tissues (Braidy et al., 2015). To our knowledge, CPS1 activity has not been linked to any age-related impairment of the urea cycle. Intracellular glutathione depletion increases protein *O*-GlcNAcylation in the skeletal muscle (Peternelj et al., 2015). Hyper-*O*-GlcNAcylation of GST family proteins may be involved in age-related oxidative stress. *O*-GlcNAcylation regulates stress-induced heat shock protein expression by regulating both HSF-1 and SP1 (Zachara et al., 2004). HSP70-GlcNAc-binding activity released by stress can protect *O*-GlcNAcylated proteins from their subsequent proteasomal degradation (Guinez et al., 2007). In our studies, four major heat shock proteins have been identified as the potential *O*-GlcNAc targets, i.e. they may be either *O*-GlcNAcylated proteins that were not mapped for the exact modification sites or the interacting proteins with *O*-GlcNAcylated proteins.

Aberrant protein *O*-GlcNAcylation plays key roles in longevity, stress, and the progression of many diseases associated with aging (Banerjee et al., 2016). Aging is characterized by cellular senescence, alteration of metabolic pathways, and susceptibility to chronic diseases (Lopez-Otin et al., 2013; Zhang et al., 2020). Persistent metabolic stress, such as hyperglycemia and glucose variability, can induce oxidative stress and increase the activity of HBP, which increases the substrate supply for *O*-GlcNAcylation (Papachristoforou et al., 2020). Reciprocally, changes in *O*-GlcNAc levels of specific proteins and sites are responsible for hepatic insulin resistance, glucose toxicity, insulin-induced *de novo* lipogenesis, necroptosis, and tissue fibrosis, contributing to the development of metabolic diseases (Zhang et al., 2014, 2019, 2021a; Banerjee et al., 2016). Recent evidence indicates that glucose flux promotes lipogenesis through liver X receptor *O*-GlcNAcylation and transcriptional activity on the promoter of sterol regulatory element-binding protein 1c (Bindesboll et al., 2015; Baldini et al., 2016; Tan et al., 2021). Here, we discovered higher levels of *O*-GlcNAcylation in aged liver and muscle compared with young tissues, suggesting a correlation between *O*-GlcNAc signaling and altered macronutrient metabolism in the aging process. Increased *O*-GlcNAc potentially contributes to adaptive and maladaptive intracellular signaling for metabolic dysfunction in aging and chronic diseases, which has not been fully addressed. Targeting overactivated *O*-GlcNAc signaling is beneficial in the diabetic heart (Prakoso et al., 2022), which may be applicable in treating age-related heart disease.

The age-associated increase of *O*-GlcNAcylation in tissues may be adaptive to defend thermal homeostasis. Hypothermia is more threatening to the elderly than to young people (Young and Lee, 1997). *O*-GlcNAcylation of p65 enhanced p65 activity and nuclear translocation, leading to the upregulation of interleukin-6, which maintains energy homeostasis and promotes cell survival in mouse skeletal muscle during cold exposure (Hu et al., 2022). Deletion of *Ogt* in brown adipocytes resulted in severe cold intolerance with decreased uncoupling protein 1 expression (Ohashi et al., 2017). We postulate that the increased levels of *O*-GlcNAc in aged BAT and muscle are likely related to metabolic heat production elicited by cold exposure and hypothermia in aging. Thus, *O*-GlcNAc may indicate the degree of age-related stress in metabolic tissues.

The OGT/OGA enzyme pair is recognized as the core of a multilevel molecular machinery that integrates stress and metabolic signals from different organelles, including mitochondria (Ong et al., 2018). The dynamics of *O*-GlcNAc cycling are determined by the availability of the donor substrate UDP-GlcNAc, protein substrates, and binding partners, as well as OGT/OGA enzyme activity. OGT and OGA themselves are subject to transcriptional and posttranslational regulation, which strikes a balance of protein *O*-GlcNAcylation (Qian et al., 2018). This paradigm may underlie the optimal metabolic adaptation to aging in a tissue-specific manner. For example, protein levels of OGT and OGA were regulated differentially in BAT (Figure 1D and F), which may reflect an adaptative response to a high level of *O*-GlcNAcylation associated with lipid accumulation (Charles et al., 2017). In the liver and muscle, where OGT and OGA are not significantly altered, increased UDP-GlcNAc levels and PTMs of OGT/OGA may lead to protein hyper-*O*-GlcNAcylation.

The liver reprograms mitochondrial metabolism to adapt to a rising flux of amino acids and fatty acids for production of glucose and ketone bodies during prolonged fasting or CR (Chimienti et al., 2021; Zhang et al., 2021b). To prevent ammonia, the urea cycle is induced. It is known that SIRT5 removes a panel of different protein acylation, i.e. acetylation, succinylation, and glutarylation, from CPS1 to sustain a high flux into the urea cycle during CR (Nakagawa et al., 2009; Park et al., 2013; Tan et al., 2014b). CR promotes amino acid catabolism through SIRT3-mediated deacetylation of ornithine transcarbamoylase from the urea cycle (Hallows et al., 2011). In our studies, dietary restriction, such as prolonged fasting and CR, unanimously decreased levels of global *O*-GlcNAcylation and *O*-GlcNAcylated CPS1, which leads to increased CPS1 enzyme activity. The intracellular colocalization of CPS1 and OGA suggests that CPS1 *O*-GlcNAcylation is reversible. The dual enzymatic activity of acyltransferase and *O*-GlcNAcase on OGA may link acetylation and de-*O*-GlcNAcylation on CPS1. These findings raise the possibility that SIRT5-mediated deacylation (including deacetylation) and OGT-mediated Ser537 *O*-GlcNAcylation may coordinately regulate CPS1 activity for optimal adaptation to dietary restriction. Collectively, these results indicate that CR reciprocally modulates various nutrient-sensing PTMs to promote amino acid catabolism. Nevertheless, it remains unknown whether and how these protein modifications crosstalk to coordinate metabolic adaptative responses. Uncovering the topology of these interactions would shed light on the precise therapeutic target that can mimic the anti-aging effects of dietary restriction.

## Materials and methods

### Animal studies

All procedures have been approved by the Institutional Animal Care and Use Committee of Yale University. Young (4-month-old) and old (24-month-old) male C57BL/6 mice and 12-month-old male C57BL/6 mice subjected to CR and age-matched control mice fed ad libitum were purchased from National Institute on Aging/National Institutes of Health. The skeletal muscle, liver, BAT, and heart were collected for biochemical analysis of *O*-GlcNAc signaling. Male OGT^flox/Y^ (5-month-old) mice were generated previously (Li et al., 2013). Recombinant adenoviruses (6×10^8^ plaque forming units to male OGT^flox/Y^ mice) were delivered by systemic tail-vein injection to mice. Three to seven days after viral infection, mice were subjected to 48 h fasting.

### Plasmids

Vectors for eukaryotic expression of the full-length and catalytically dead OGT were described previously (Chen et al., 2013). pSG5-hCPS1-FLAG plasmid was kindly offered by Matthew D. Hirschey (Duke University School of Medicine). Point mutants of CPS1 were generated with the QuikChange XL II Site-Directed Mutagenesis Kit (Stratagene). p3Flag/2Myc-OGA (WT), p3Flag/2Myc-OGA (D177N), and p3Flag/2Myc-OGA (Y891F) were kindly offered by Andrew J. Paterson (Chalmers University of Technology), and the fragments of NCOAT were subcloned into pAdTrack-CMV plasmids. Overexpression adenovirus constructs were established using the AdEasy system (Luo et al., 2007). Adenoviruses were amplified in HEK293 cells and purified using the kit from Virapur.

### Cell culture

HepG2, HeLa, and HEK293T cells were cultured in Dulbecco's modified Eagle's medium (DMEM) with 10% fetal bovine serum (FBS). Primary hepatocytes were isolated by Yale Liver Center Core Facility and plated in DMEM with 10% FBS, 2 mM sodium pyruvate, 1 mM dexamethasome, and 0.1 mM insulin on collagen I-coated plates. HepG2 and HEK293T cells were transfected with FuGENE HD (Roche). Primary hepatocytes were infected with adenovirus in medium containing 0.5% bovine serum album (BSA). Treatment with different glucose levels was performed with no glucose DMEM plus 10% dialyzed FBS and various concentrations of glucose for 8 h. The OGA inhibitor PUGNAc (10 mM, 16 h) was used as indicated. HeLa cells stably transfected with pTRE2pur-3Flag/2Myc-OGA and pTet-Off plasmids were cultured in DMEM with 10% FBS and doxycycline (1 µg/ml).

### Antibodies, IP, and western blotting

Anti-Flag (F3165) and anti-β-actin (A5441) antibodies were from Sigma-Aldrich. Anti-CPS1 (ab3682), anti-*O*-GlcNAc (RL-2, ab2739), anti-OGT (ab50270), and anti-OGA/meningioma expressed antigen 5 (MGEA5) (ab68522) antibodies were from Abcam. Anti-Myc (9E10, sc-40) antibody was from Santa Cruz Biotechnology. Procedures for IP and immunoblotting (IB) assays were described previously (Ruan et al., 2012).

### Immunostaining and confocal microscopy

HeLa cells were fixed with paraformaldehyde for 10 min on ice. After washing with phosphate-buffered saline (PBS), pH 7.4, cells were incubated with 0.2% Triton X-100 for 5 min and blocked with 10% goat serum at room temperature for 30 min. Thereafter, cells were incubated overnight at 4°C with rabbit anti-CPS1 and mouse anti-MGEA. Anti-rabbit Alexa Fluor^®^488-conjugated secondary antibody (Molecular Probes) and anti-mouse Alexa Fluor^®^537-conjugated secondary antibody were used. Nuclei were stained with DAPI. Antibody staining was detected at an emission of 500–530, 550–600, and 640–720 nm after excitation at 488, 543, and 630 nm, respectively. An oil immersion objective (Zeiss ×63/1.4 numerical aperture) was used. The image showed CPS1 in green, OGA in red, and nuclei in blue.

### Mitochondrion isolation

Mitochondria were isolated from the livers of OGT knockout mice as described previously (Wieckowski et al., 2009). Briefly, livers were homogenized with a glass-Teflon Potter homogenizer in medium containing 225 mM mannitol, 75 mM sucrose, 0.5% BSA, 0.5 mM ethylene glycol-bis(β-aminoethyl ether)-*N,N,N*′,*N*′-tetraacetic acid (EGTA), and 30 mM Tris–HCl (pH 7.4). The mitochondria were isolated in a low-speed centrifuge (740× *g*/min) and washed twice in the same medium without EGTA. A high-speed centrifuge (9000× *g*/min) precipitated the purified mitochondria. Then, the mitochondria were resuspended in mitochondria resuspending buffer containing 250 mM mannitol, 5 mM HEPES (pH 7.4), and 0.5 mM EGTA. The mitochondrial protein concentration was measured using a protein DC assay kit (Bio Rad).

### CPS1 enzyme activity assay

CPS1 enzyme activity was assessed by measuring converted citrulline with a colorimetric method (Nakagawa et al., 2009). Briefly, mitochondria were incubated in CPS1 assay buffer (50 mM Tris–HCl, pH 7.6, 50 mM KHCO_3_, 35 mM (NH_4_)_2_SO_4_, 15 mM MgSO_4_, 10 mM ATP, 5 mM L-ornithine, 10 mM *N*-acetylglutamate, and 2 unit/ml ornithine transcarbamylase) for 10 min at 37°C. The reaction was terminated by adding 8% TCA. A color development solution was prepared just before the assay by mixing 400 μl solution 1 (0.74 g antipyrine, 6.25 ml 8% ferric ammonium sulfate, 50 ml 95% H_2_SO4, and 50 ml 85% H_3_PO_4_ in 200 ml) and 200 μl solution 2 (0.4 g diacetyl monoxime and 7.5 g NaCl in 100 ml) for each assay. After 100 μl reactant and 600 μl color development solution were mixed, tubes were boiled at 100°C for 15 min and then transferred to water at room temperature for cooling. The absorbance at 464 nm in a 1-cm light path was measured. The relation between citrulline concentration and light absorbance obeys Beer's law, with a millimolar extinction coefficient of 37.8. Activity was calculated by unit definition as fellows (unit/mg protein = OD × 0.8 × 3.5 × 6/37.8 mg protein).

### Ammonia assay

Serum ammonia was detected with an Ammonia Colorimetric Assay Kit (Biovision, K370-100) as per the manual. The samples and ammonium chloride standard were added to 96-well plate, bringing the volume to 50 μl/well with assay buffer. Then, 50 μl of the reaction mix was added to each well. The reaction was incubated for 60 min at 37°C and protected from light. The OD 570 nm was measured in a micro plate reader. NH_4_Cl concentrations in the samples were calculated according to the NH_4_Cl standard curve.

### Statistical analyses

Results are shown as mean ± SEM. The comparisons between two groups were carried out using two-tailed unpaired Student's *t*-test. For multiple-group comparisons, one-way analysis of variance was used for determining no differences among the group means. *Post hoc* comparisons were adjusted using Bonferroni corrections. Statistical analysis was accepted as significant if the *P*-value was <0.05.

## Supplementary Material

mjac016_Supplemental_File

## References

[bib1] Alteen M.G. , Tan H.Y., Vocadlo D.J. (2021). Monitoring and modulating O-GlcNAcylation: assays and inhibitors of O-GlcNAc processing enzymes. Curr. Opin. Struct. Biol. 68, 157–165. 33535148 10.1016/j.sbi.2020.12.008

[bib2] Baldini S.F. , Wavelet C., Hainault I. et al. (2016). The nutrient-dependent O-GlcNAc modification controls the expression of liver fatty acid synthase. J. Mol. Biol. 428, 3295–3304. 27185461 10.1016/j.jmb.2016.04.035

[bib3] Banerjee P.S. , Lagerlof O., Hart G.W. (2016). Roles of O-GlcNAc in chronic diseases of aging. Mol. Aspects Med. 51, 1–15. 27259471 10.1016/j.mam.2016.05.005

[bib4] Banerjee P.S. , Ma J., Hart G.W. (2015). Diabetes-associated dysregulation of O-GlcNAcylation in rat cardiac mitochondria. Proc. Natl Acad. Sci. USA 112, 6050–6055. 25918408 10.1073/pnas.1424017112 PMC4434690

[bib5] Bindesboll C. , Fan Q., Norgaard R.C. et al. (2015). Liver X receptor regulates hepatic nuclear O-GlcNAc signaling and carbohydrate responsive element-binding protein activity. J. Lipid Res. 56, 771–785. 25724563 10.1194/jlr.M049130 PMC4373736

[bib6] Braidy N. , Poljak A., Grant R. et al. (2015). Differential expression of sirtuins in the aging rat brain. Front. Cell. Neurosci. 9, 167. 26005404 10.3389/fncel.2015.00167 PMC4424846

[bib7] Bratic I. , Trifunovic A. (2010). Mitochondrial energy metabolism and ageing. Biochim. Biophys. Acta 1797, 961–967. 20064485 10.1016/j.bbabio.2010.01.004

[bib8] Cao W. , Cao J., Huang J. et al. (2013). Discovery and confirmation of O-GlcNAcylated proteins in rat liver mitochondria by combination of mass spectrometry and immunological methods. PLoS One 8, e76399. 24098488 10.1371/journal.pone.0076399 PMC3788734

[bib9] Charles K.N. , Li M.D., Engin F. et al. (2017). Uncoupling of metabolic health from longevity through genetic alteration of adipose tissue lipid-binding proteins. Cell Rep. 21, 393–402. 29020626 10.1016/j.celrep.2017.09.051 PMC5682597

[bib10] Chatham J.C. , Zhang J., Wende A.R. (2021). Role of O-linked N-acetylglucosamine protein modification in cellular (patho)physiology. Physiol. Rev. 101, 427–493. 32730113 10.1152/physrev.00043.2019 PMC8428922

[bib11] Chen Q. , Chen Y., Bian C. et al. (2013). TET2 promotes histone O-GlcNAcylation during gene transcription. Nature 493, 561–564. 23222540 10.1038/nature11742 PMC3684361

[bib12] Chimienti G. , Picca A., Fracasso F. et al. (2021). The age-sensitive efficacy of calorie restriction on mitochondrial biogenesis and mtDNA damage in rat liver. Int. J. Mol. Sci. 22, 1665. 33562258 10.3390/ijms22041665 PMC7915472

[bib13] Crouser E.D. , Julian M.W., Huff J.E. et al. (2006). Carbamoyl phosphate synthase-1: a marker of mitochondrial damage and depletion in the liver during sepsis. Crit. Care Med. 34, 2439–2446. 16791110 10.1097/01.CCM.0000230240.02216.21

[bib14] Dhahbi J.M. , Mote P.L., Wingo J. et al. (2001). Caloric restriction alters the feeding response of key metabolic enzyme genes. Mech. Ageing Dev. 122, 1033–1048. 11389922 10.1016/s0047-6374(01)00230-5

[bib15] Du J. , Zhou Y., Su X. et al. (2011). Sirt5 is a NAD-dependent protein lysine demalonylase and desuccinylase. Science 334, 806–809. 22076378 10.1126/science.1207861 PMC3217313

[bib16] Easlon E. , Tsang F., Dilova I. et al. (2007). The dihydrolipoamide acetyltransferase is a novel metabolic longevity factor and is required for calorie restriction-mediated life span extension. J. Biol. Chem. 282, 6161–6171. 17200108 10.1074/jbc.M607661200 PMC2440684

[bib17] Fulop N. , Feng W., Xing D. et al. (2008). Aging leads to increased levels of protein O-linked N-acetylglucosamine in heart, aorta, brain and skeletal muscle in Brown-Norway rats. Biogerontology 9, 139–151. 18185980 10.1007/s10522-007-9123-5 PMC2810282

[bib18] Gao A.W. , Canto C., Houtkooper R.H. (2014). Mitochondrial response to nutrient availability and its role in metabolic disease. EMBO Mol. Med. 6, 580–589. 24623376 10.1002/emmm.201303782 PMC4023882

[bib19] Green C.L. , Lamming D.W., Fontana L. (2022). Molecular mechanisms of dietary restriction promoting health and longevity. Nat. Rev. Mol. Cell Biol. 23, 56–73. 34518687 10.1038/s41580-021-00411-4 PMC8692439

[bib20] Guinez C. , Mir A.M., Leroy Y. et al. (2007). Hsp70-GlcNAc-binding activity is released by stress, proteasome inhibition, and protein misfolding. Biochem. Biophys. Res. Commun. 361, 414–420. 17645866 10.1016/j.bbrc.2007.07.020

[bib21] Hallows W.C. , Yu W., Smith B.C. et al. (2011). Sirt3 promotes the urea cycle and fatty acid oxidation during dietary restriction. Mol. Cell 41, 139–149. 21255725 10.1016/j.molcel.2011.01.002 PMC3101115

[bib22] Hart G.W. (2019). Nutrient regulation of signaling and transcription. J. Biol. Chem. 294, 2211–2231. 30626734 10.1074/jbc.AW119.003226 PMC6378989

[bib23] Harwood K.R. , Hanover J.A. (2014). Nutrient-driven O-GlcNAc cycling—think globally but act locally. J. Cell Sci. 127, 1857–1867. 24762810 10.1242/jcs.113233 PMC4004970

[bib24] Hofer A. , Wenz T. (2014). Post-translational modification of mitochondria as a novel mode of regulation. Exp. Gerontol. 56, 202–220. 24632076 10.1016/j.exger.2014.03.006

[bib25] Hu Y. , Liu Y., Yang Y. et al. (2022). OGT upregulates myogenic IL-6 by mediating O-GlcNAcylation of p65 in mouse skeletal muscle under cold exposure. J. Cell. Physiol. 237, 1341–1352. 34668190 10.1002/jcp.30612

[bib26] Hu Y. , Suarez J., Fricovsky E. et al. (2009). Increased enzymatic O-GlcNAcylation of mitochondrial proteins impairs mitochondrial function in cardiac myocytes exposed to high glucose. J. Biol. Chem. 284, 547–555. 19004814 10.1074/jbc.M808518200 PMC2610513

[bib27] Jahangir Z. , Ahmad W., Shabbiri K. (2014). Alternate phosphorylation/O-GlcNAc modification on human insulin IRSs: a road towards impaired insulin signaling in Alzheimer and diabetes. Adv. Bioinform. 2014, 324753. 10.1155/2014/324753 PMC4281456 25580119

[bib28] Johnsen V.L. , Belke D.D., Hughey C.C. et al. (2013). Enhanced cardiac protein glycosylation (O-GlcNAc) of selected mitochondrial proteins in rats artificially selected for low running capacity. Physiol. Genomics 45, 17–25. 23132757 10.1152/physiolgenomics.00111.2012 PMC3544485

[bib29] Jozwiak P. , Ciesielski P., Zakrzewski P.K. et al. (2021). Mitochondrial O-GlcNAc transferase interacts with and modifies many proteins and its up-regulation affects mitochondrial function and cellular energy homeostasis. Cancers 13, 2956. 34204801 10.3390/cancers13122956 PMC8231590

[bib30] Jozwiak P. , Forma E., Brys M. et al. (2014). O-GlcNAcylation and metabolic reprograming in cancer. Front. Endocrinol. 5, 145. 10.3389/fendo.2014.00145 PMC4158873 25250015

[bib31] Lee J. , Kim K.Y., Lee J. et al. (2010). Regulation of Dauer formation by O-GlcNAcylation in Caenorhabditis elegans. J. Biol. Chem. 285, 2930–2939. 19940149 10.1074/jbc.M109.022665 PMC2823417

[bib32] Li M.D. , Ruan H.B., Hughes M.E. et al. (2013). O-GlcNAc signaling entrains the circadian clock by inhibiting BMAL1/CLOCK ubiquitination. Cell Metab. 17, 303–310. 23395176 10.1016/j.cmet.2012.12.015 PMC3647362

[bib33] Li M.D. , Vera N.B., Yang Y. et al. (2018). Adipocyte OGT governs diet-induced hyperphagia and obesity. Nat. Commun. 9, 5103. 30504766 10.1038/s41467-018-07461-x PMC6269424

[bib34] Liu K. , Paterson A.J., Chin E. et al. (2000). Glucose stimulates protein modification by O-linked GlcNAc in pancreatic beta cells: linkage of O-linked GlcNAc to beta cell death. Proc. Natl Acad. Sci. USA 97, 2820–2825. 10717000 10.1073/pnas.97.6.2820 PMC16013

[bib35] Lopez-Lluch G. , Hunt N., Jones B. et al. (2006). Calorie restriction induces mitochondrial biogenesis and bioenergetic efficiency. Proc. Natl Acad. Sci. USA 103, 1768–1773. 16446459 10.1073/pnas.0510452103 PMC1413655

[bib36] Lopez-Otin C. , Blasco M.A., Partridge L. et al. (2013). The hallmarks of aging. Cell 153, 1194–1217. 23746838 10.1016/j.cell.2013.05.039 PMC3836174

[bib37] Luo J. , Deng Z.L., Luo X. et al. (2007). A protocol for rapid generation of recombinant adenoviruses using the AdEasy system. Nat. Protoc. 2, 1236–1247. 17546019 10.1038/nprot.2007.135

[bib38] Ma J. , Wu C., Hart G.W. (2021). Analytical and biochemical perspectives of protein O-GlcNAcylation. Chem. Rev. 121, 1513–1581. 33416322 10.1021/acs.chemrev.0c00884

[bib39] Marotta N.P. , Cherwien C.A., Abeywardana T. et al. (2012). O-GlcNAc modification prevents peptide-dependent acceleration of alpha-synuclein aggregation. ChemBioChem 13, 2665–2670. 23143740 10.1002/cbic.201200478

[bib40] Nakagawa T. , Lomb D.J., Haigis M.C. et al. (2009). SIRT5 deacetylates carbamoyl phosphate synthetase 1 and regulates the urea cycle. Cell 137, 560–570. 19410549 10.1016/j.cell.2009.02.026 PMC2698666

[bib41] Ohashi N. , Morino K., Ida S. et al. (2017). Pivotal role of O-GlcNAc modification in cold-induced thermogenesis by brown adipose tissue through mitochondrial biogenesis. Diabetes 66, 2351–2362. 28637651 10.2337/db16-1427

[bib42] Ong Q. , Han W., Yang X. (2018). O-GlcNAc as an integrator of signaling pathways. Front. Endocrinol. 9, 599. 10.3389/fendo.2018.00599 PMC6234912 30464755

[bib43] Papachristoforou E. , Lambadiari V., Maratou E. et al. (2020). Association of glycemic indices (hyperglycemia, glucose variability, and hypoglycemia) with oxidative stress and diabetic complications. J. Diabetes Res. 2020, 1. 10.1155/2020/7489795 PMC7585656 33123598

[bib44] Park J. , Chen Y., Tishkoff D.X. et al. (2013). SIRT5-mediated lysine desuccinylation impacts diverse metabolic pathways. Mol. Cell 50, 919–930. 23806337 10.1016/j.molcel.2013.06.001 PMC3769971

[bib45] Peternelj T.T. , Marsh S.A., Strobel N.A. et al. (2015). Glutathione depletion and acute exercise increase O-GlcNAc protein modification in rat skeletal muscle. Mol. Cell. Biochem. 400, 265–275. 25416863 10.1007/s11010-014-2283-0

[bib46] Plubell D.L. , Wilmarth P.A., Zhao Y. et al. (2017). Extended multiplexing of tandem mass tags (TMT) labeling reveals age and high fat diet specific proteome changes in mouse epididymal adipose tissue. Mol. Cell. Proteomics 16, 873–890. 28325852 10.1074/mcp.M116.065524 PMC5417827

[bib47] Prakoso D. , Lim S.Y., Erickson J.R. et al. (2022). Fine-tuning the cardiac O-GlcNAcylation regulatory enzymes governs the functional and structural phenotype of the diabetic heart. Cardiovasc. Res. 118, 212–225. 33576380 10.1093/cvr/cvab043

[bib48] Qian K. , Wang S., Fu M. et al. (2018). Transcriptional regulation of O-GlcNAc homeostasis is disrupted in pancreatic cancer. J. Biol. Chem. 293, 13989–14000. 30037904 10.1074/jbc.RA118.004709 PMC6130940

[bib49] Rahman M.M. , Stuchlick O., El-Karim E.G. et al. (2010). Intracellular protein glycosylation modulates insulin mediated lifespan in C. elegans. Aging 2, 678–690. 20952811 10.18632/aging.100208 PMC2993798

[bib50] Ruan H.B. , Han X., Li M.D. et al. (2012). O-GlcNAc transferase/host cell factor C1 complex regulates gluconeogenesis by modulating PGC-1α stability. Cell Metab. 16, 226–237. 22883232 10.1016/j.cmet.2012.07.006 PMC3480732

[bib51] Ruan H.B. , Ma Y., Torres S. et al. (2017). Calcium-dependent O-GlcNAc signaling drives liver autophagy in adaptation to starvation. Genes Dev. 31, 1655–1665. 28903979 10.1101/gad.305441.117 PMC5647936

[bib52] Ruan H.B. , Singh J.P., Li M.D. et al. (2013). Cracking the O-GlcNAc code in metabolism. Trends Endocrinol. Metab. 24, 301–309. 23647930 10.1016/j.tem.2013.02.002 PMC3783028

[bib53] Ryu D. , Jo Y.S., Lo Sasso G. et al. (2014). A SIRT7-dependent acetylation switch of GABPβ1 controls mitochondrial function. Cell Metab. 20, 856–869. 25200183 10.1016/j.cmet.2014.08.001

[bib54] Singh J.P. , Qian K., Lee J.S. et al. (2020). O-GlcNAcase targets pyruvate kinase M2 to regulate tumor growth. Oncogene 39, 560–573. 31501520 10.1038/s41388-019-0975-3 PMC7107572

[bib55] Stephen H.M. , Adams T.M., Wells L. (2021). Regulating the regulators: mechanisms of substrate selection of the O-GlcNAc cycling enzymes OGT and OGA. Glycobiology 31, 724–733. 33498085 10.1093/glycob/cwab005 PMC8351506

[bib56] Tan E.P. , Villar M.T., Lezi E. et al. (2014a). Altering O-linked β-N-acetylglucosamine cycling disrupts mitochondrial function. J. Biol. Chem. 289, 14719–14730. 24713701 10.1074/jbc.M113.525790 PMC4031527

[bib57] Tan M. , Peng C., Anderson K.A. et al. (2014b). Lysine glutarylation is a protein posttranslational modification regulated by SIRT5. Cell Metab. 19, 605–617. 24703693 10.1016/j.cmet.2014.03.014 PMC4108075

[bib58] Tan W. , Jiang P., Zhang W. et al. (2021). Posttranscriptional regulation of de novo lipogenesis by glucose-induced O-GlcNAcylation. Mol. Cell 81, 1890–1904.e7. 33657401 10.1016/j.molcel.2021.02.009

[bib59] Toleman C. , Paterson A.J., Whisenhunt T.R. et al. (2004). Characterization of the histone acetyltransferase (HAT) domain of a bifunctional protein with activable O-GlcNAcase and HAT activities. J. Biol. Chem. 279, 53665–53673. 15485860 10.1074/jbc.M410406200

[bib60] Wheatley E.G. , Albarran E., White C.W., 3rd et al. (2019). Neuronal O-GlcNAcylation improves cognitive function in the aged mouse brain. Curr. Biol. 29, 3359–3369.e4. 31588002 10.1016/j.cub.2019.08.003 PMC7199460

[bib61] Wieckowski M.R. , Giorgi C., Lebiedzinska M. et al. (2009). Isolation of mitochondria-associated membranes and mitochondria from animal tissues and cells. Nat. Protoc. 4, 1582–1590. 19816421 10.1038/nprot.2009.151

[bib62] Wilson K.A. , Chamoli M., Hilsabeck T.A. et al. (2021). Evaluating the beneficial effects of dietary restrictions: a framework for precision nutrigeroscience. Cell Metab. 33, 2142–2173. 34555343 10.1016/j.cmet.2021.08.018 PMC8845500

[bib63] Yang X. , Qian K. (2017). Protein O-GlcNAcylation: emerging mechanisms and functions. Nat. Rev. Mol. Cell Biol. 18, 452–465. 28488703 10.1038/nrm.2017.22 PMC5667541

[bib64] Yi W. , Clark P.M., Mason D.E. et al. (2012). Phosphofructokinase 1 glycosylation regulates cell growth and metabolism. Science 337, 975–980. 22923583 10.1126/science.1222278 PMC3534962

[bib65] Young A.J. , Lee D.T. (1997). Aging and human cold tolerance. Exp. Aging Res. 23, 45–67. 9049612 10.1080/03610739708254026

[bib66] Yuzwa S.A. , Cheung A.H., Okon M. et al. (2014). O-GlcNAc modification of tau directly inhibits its aggregation without perturbing the conformational properties of tau monomers. J. Mol. Biol. 426, 1736–1752. 24444746 10.1016/j.jmb.2014.01.004

[bib67] Zachara N.E. , Hart G.W. (2004). O-GlcNAc a sensor of cellular state: the role of nucleocytoplasmic glycosylation in modulating cellular function in response to nutrition and stress. Biochim. Biophys. Acta 1673, 13–28. 15238246 10.1016/j.bbagen.2004.03.016

[bib68] Zachara N.E. , O'Donnell N., Cheung W.D. et al. (2004). Dynamic O-GlcNAc modification of nucleocytoplasmic proteins in response to stress. A survival response of mammalian cells. J. Biol. Chem. 279, 30133–30142. 15138254 10.1074/jbc.M403773200

[bib69] Zhang B. , Lapenta K., Wang Q. et al. (2021a). Trefoil factor 2 secreted from damaged hepatocytes activates hepatic stellate cells to induce fibrogenesis. J. Biol. Chem. 297, 100887. 34146542 10.1016/j.jbc.2021.100887 PMC8267550

[bib70] Zhang B. , Li M.D., Yin R. et al. (2019). O-GlcNAc transferase suppresses necroptosis and liver fibrosis. JCI Insight 4, e127709. 10.1172/jci.insight.127709 PMC6948774 31672932

[bib71] Zhang K. , Yin R., Yang X. (2014). O-GlcNAc: a bittersweet switch in liver. Front. Endocrinol. 5, 221. 10.3389/fendo.2014.00221 PMC4269194 25566193

[bib72] Zhang W. , Qu J., Liu G.H. et al. (2020). The ageing epigenome and its rejuvenation. Nat. Rev. Mol. Cell Biol. 21, 137–150. 32020082 10.1038/s41580-019-0204-5

[bib73] Zhang X. , Gao T., Deng S. et al. (2021b). Fasting induces hepatic lipid accumulation by stimulating peroxisomal dicarboxylic acid oxidation. J. Biol. Chem. 296, 100622. 33811861 10.1016/j.jbc.2021.100622 PMC8102918

