# Supplementary material for: Regulation of the urea cycle by CPS1 O-GlcNAcylation in response to dietary restriction and aging
Source: J Mol Cell Biol. 2022 Mar 14;14(3):mjac016. doi: 10.1093/jmcb/mjac016 (PMC9254885; doi:10.1093/jmcb/mjac016)
Supplement: mjac016_Supplemental_File [file mjac016_supplemental_file.zip › Supplementary Materials.pptx]

## Slide 1
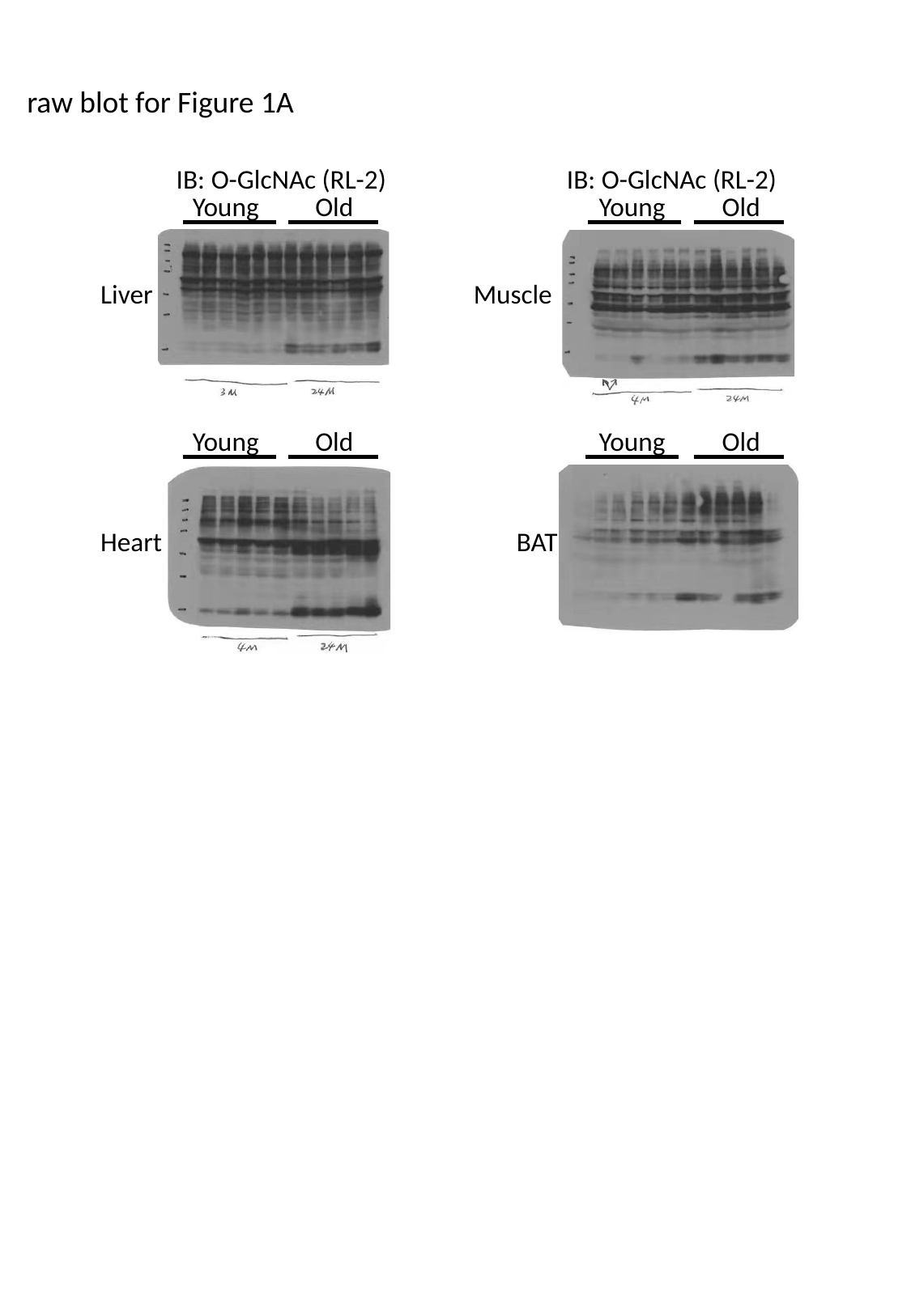

raw blot for Figure 1A
IB: O-GlcNAc (RL-2)
IB: O-GlcNAc (RL-2)
Young
Old
Young
Old
Liver
Muscle
Young
Old
Young
Old
Heart
BAT

## Slide 2
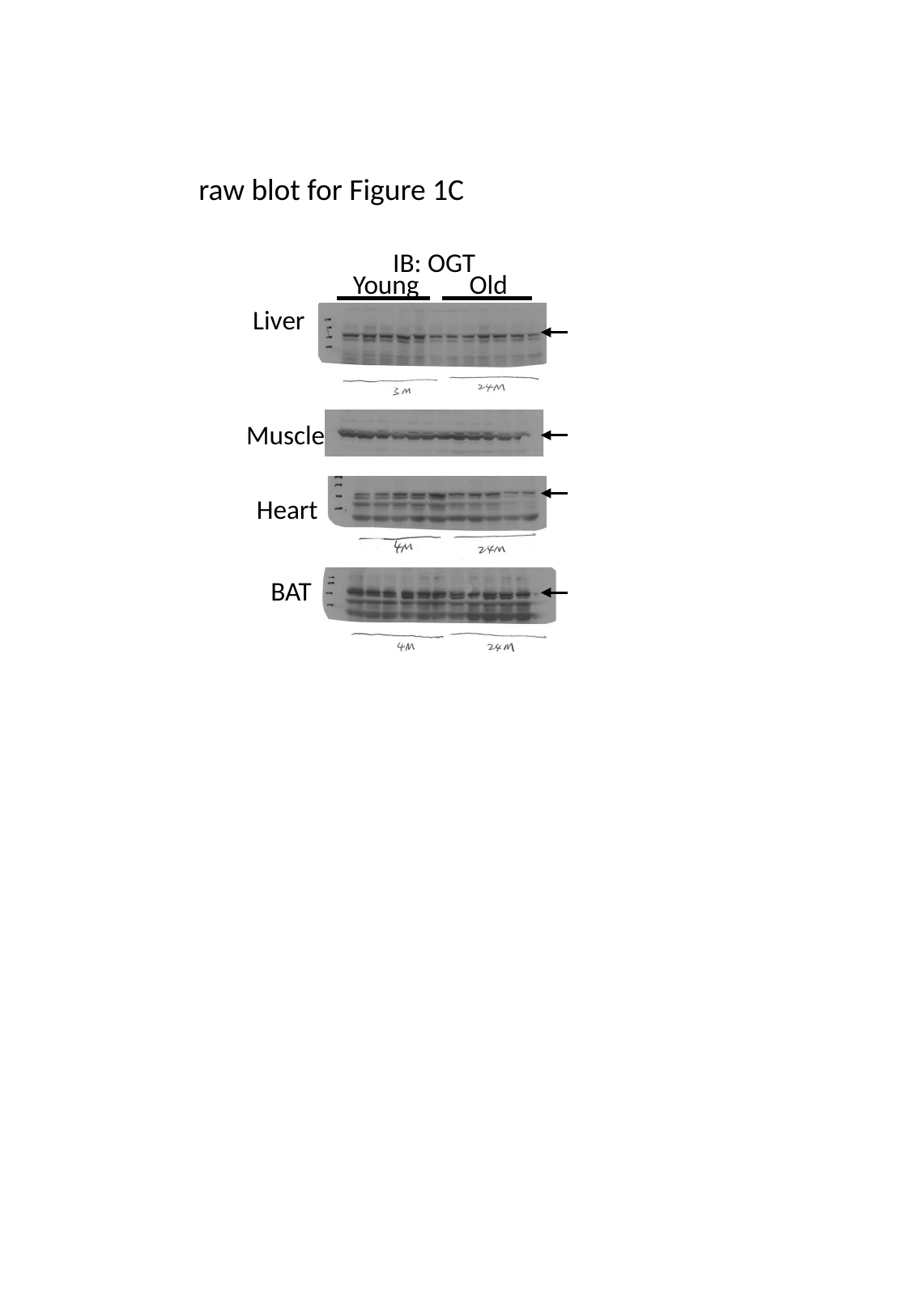

raw blot for Figure 1C
IB: OGT
Young
Old
Liver
Muscle
Heart
BAT

## Slide 3
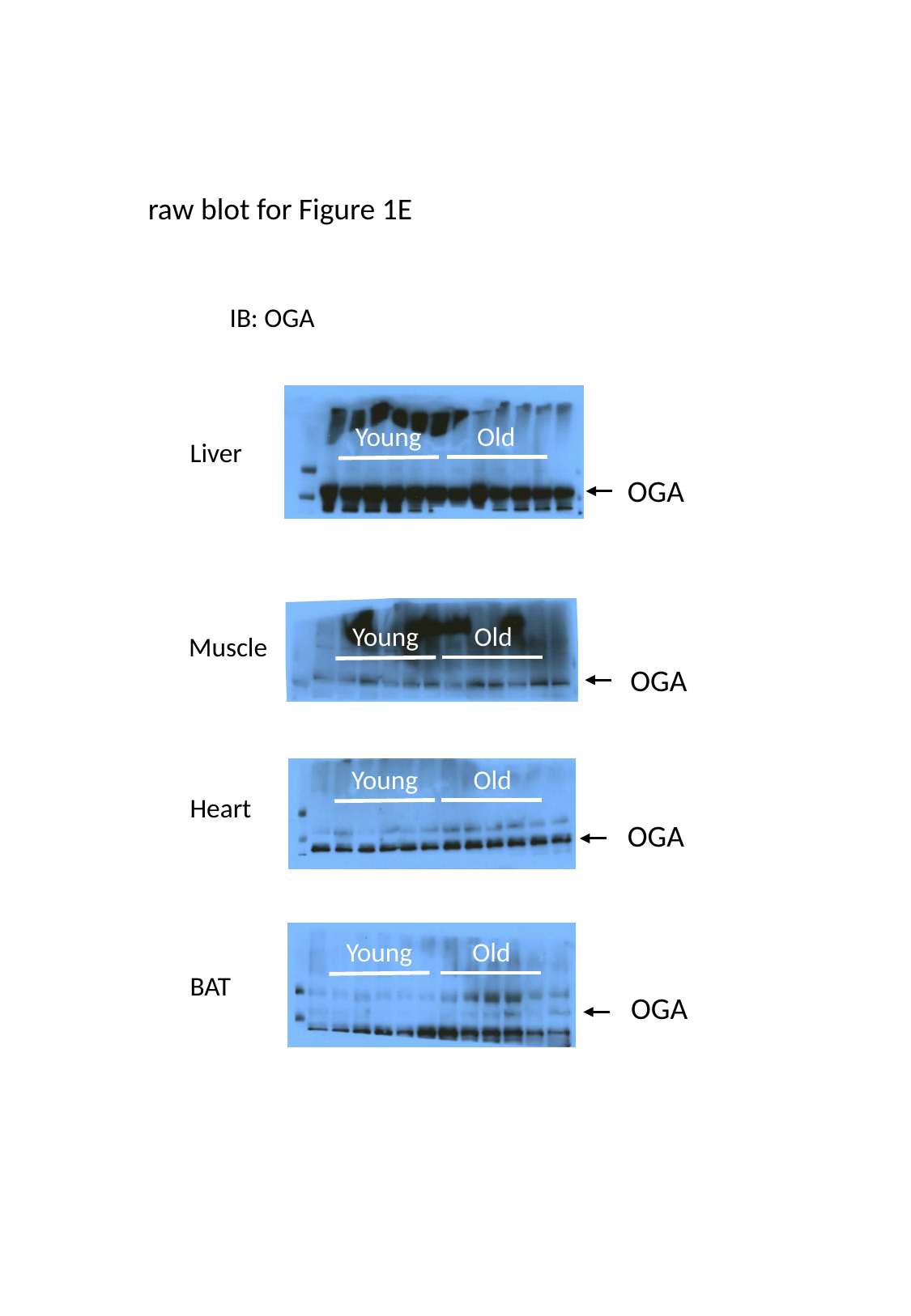

raw blot for Figure 1E
IB: OGA
Young
Old
Liver
OGA
Young
Old
Muscle
OGA
Young
Old
Heart
OGA
Young
Old
BAT
OGA

## Slide 4
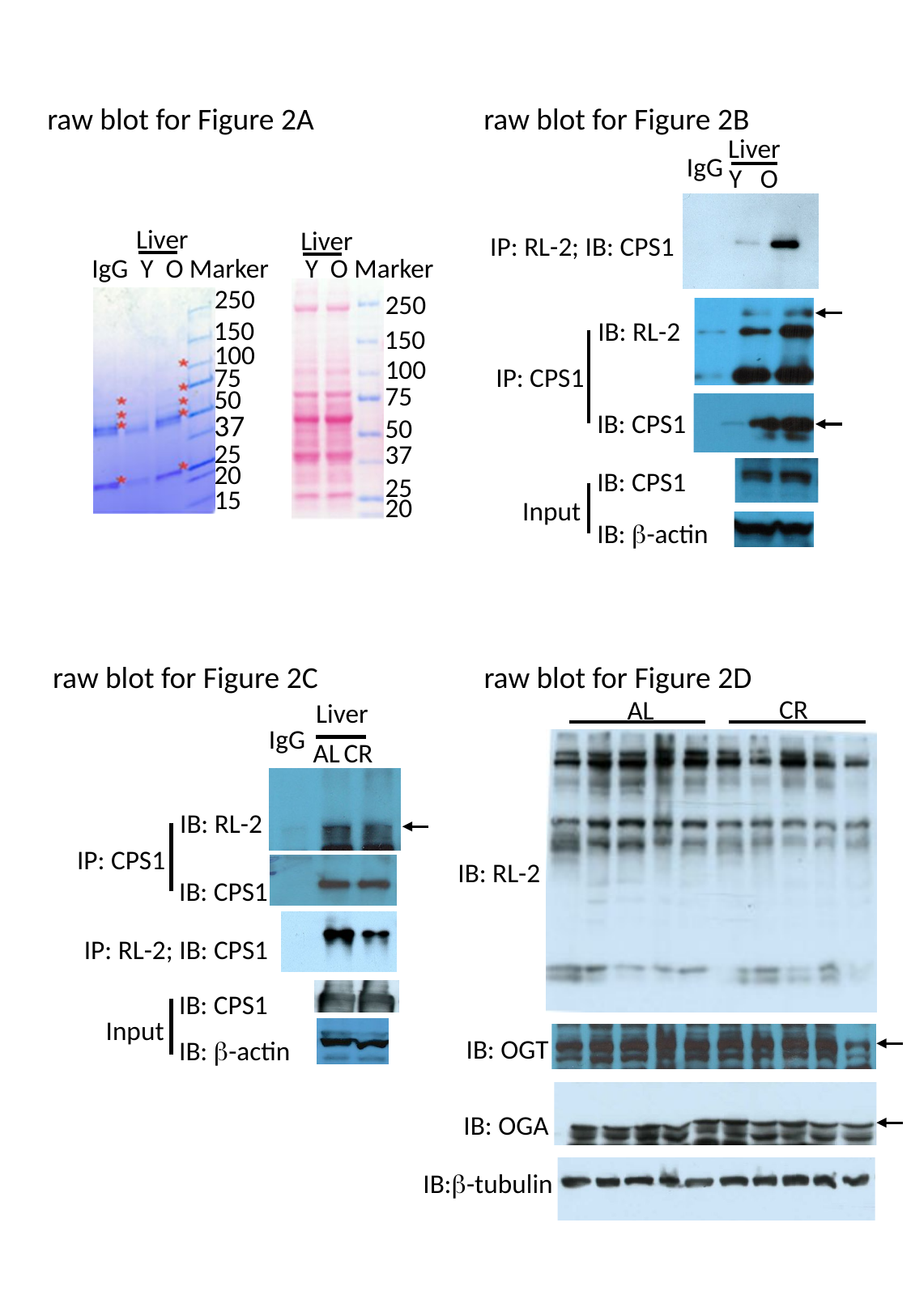

raw blot for Figure 2A
raw blot for Figure 2B
Liver
IgG
 Y O
Liver
Liver
IgG Y O Marker
Y O Marker
250
250
150
100
75
50
37
25
20
150
100
75
50
37
25
20
15
IP: RL-2; IB: CPS1
IB: RL-2
IP: CPS1
IB: CPS1
IB: CPS1
Input
IB: b-actin
raw blot for Figure 2C
raw blot for Figure 2D
CR
 AL
Liver
 IgG
 AL
CR
IB: RL-2
IP: CPS1
IB: RL-2
IB: CPS1
IP: RL-2; IB: CPS1
IB: CPS1
Input
IB: OGT
IB: b-actin
IB: OGA
IB:b-tubulin

## Slide 5
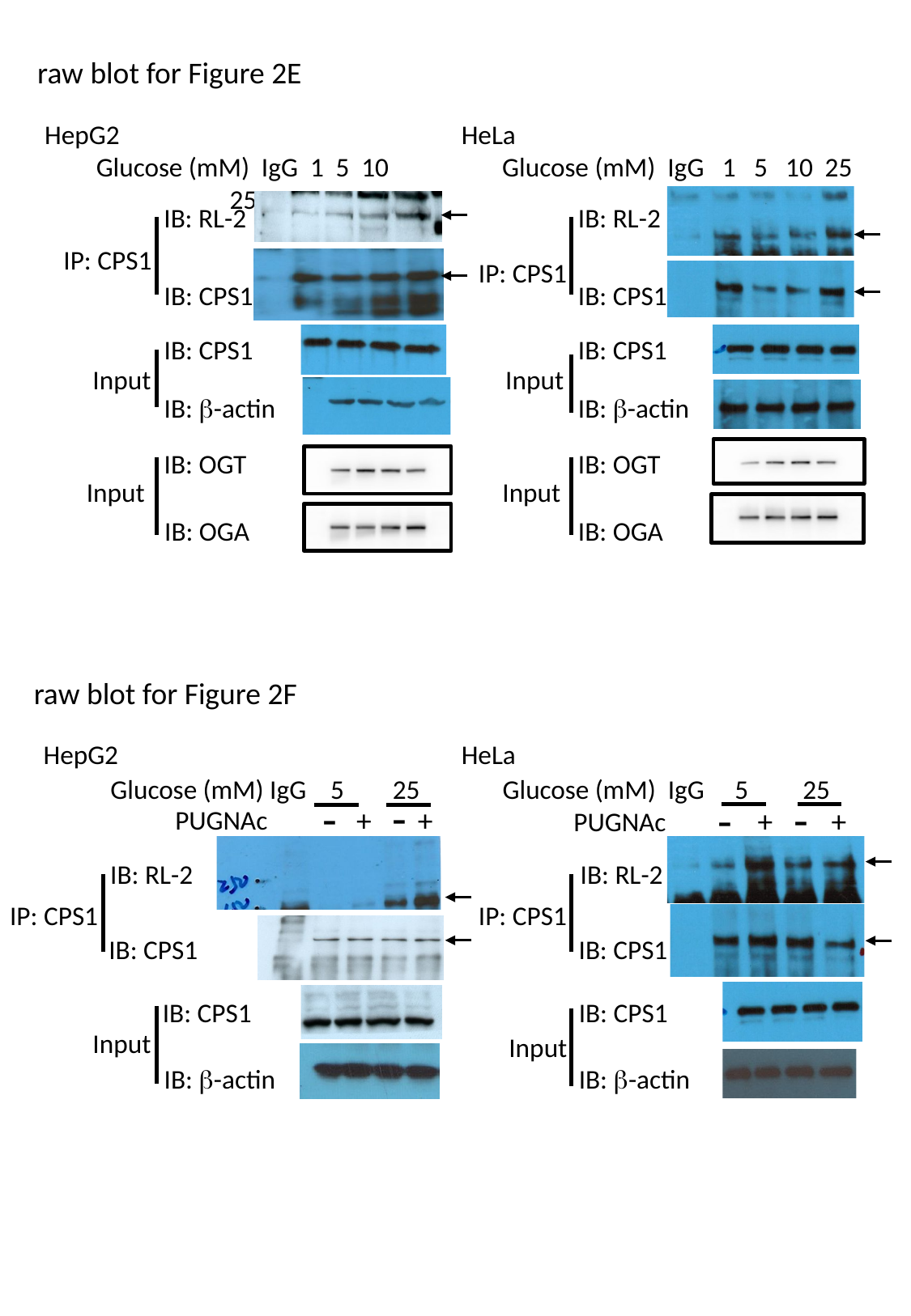

raw blot for Figure 2E
HepG2
HeLa
Glucose (mM) IgG 1 5 10	 25
Glucose (mM) IgG 1 5 10 25
IB: RL-2
IB: RL-2
IP: CPS1
IP: CPS1
IB: CPS1
IB: CPS1
IB: CPS1
IB: CPS1
Input
Input
IB: b-actin
IB: b-actin
IB: OGT
IB: OGT
Input
Input
IB: OGA
IB: OGA
raw blot for Figure 2F
HepG2
HeLa
 Glucose (mM) IgG 5 25
Glucose (mM) IgG 5 25
-
-
+
+
PUGNAc
-
-
+
+
PUGNAc
IB: RL-2
IB: RL-2
IP: CPS1
IP: CPS1
IB: CPS1
IB: CPS1
IB: CPS1
IB: CPS1
Input
Input
IB: b-actin
IB: b-actin

## Slide 6
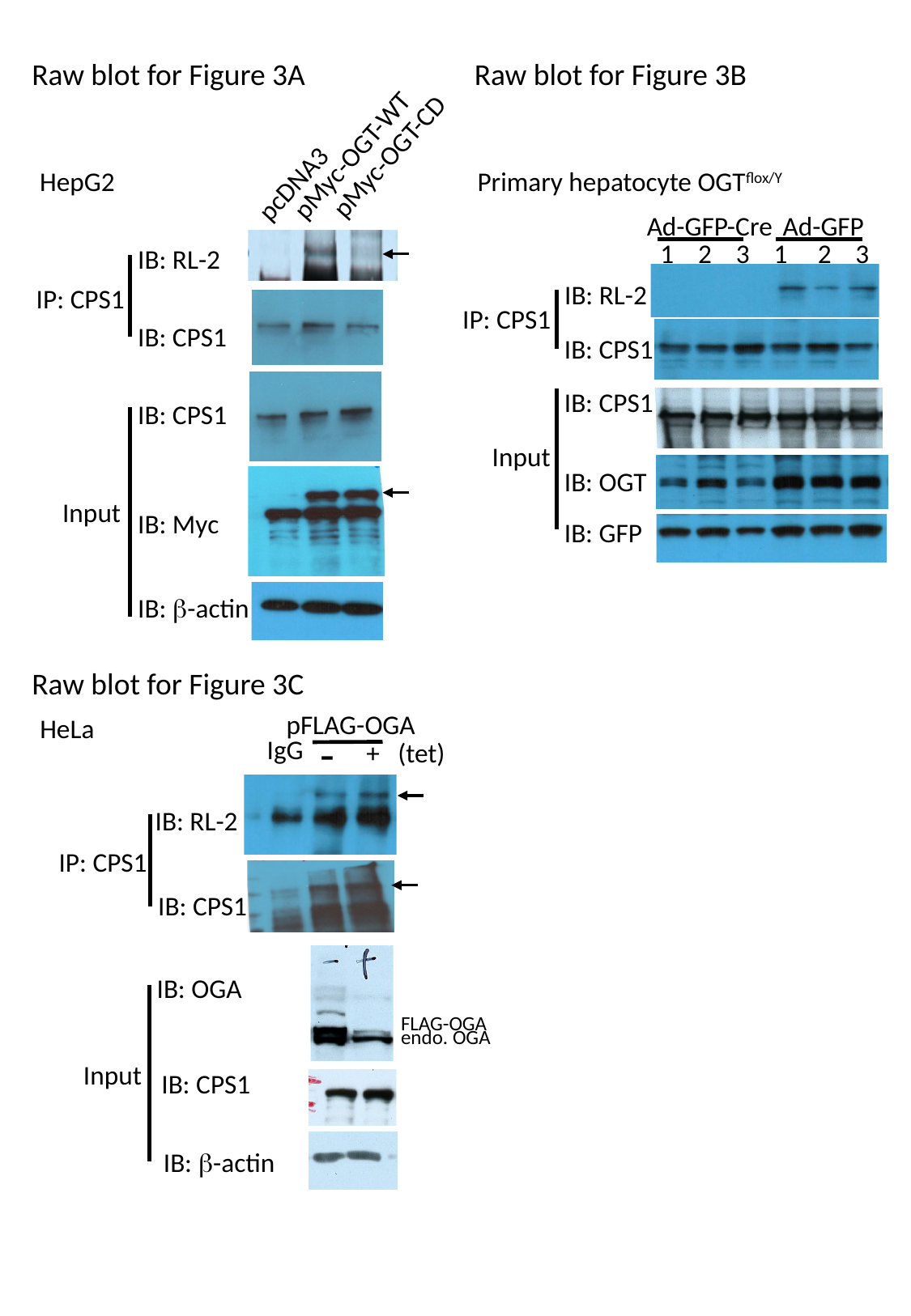

Raw blot for Figure 3A
Raw blot for Figure 3B
pMyc-OGT-WT
pMyc-OGT-CD
HepG2
Primary hepatocyte OGTflox/Y
pcDNA3
Ad-GFP-Cre
Ad-GFP
1 2 3 1 2 3
IB: RL-2
IB: RL-2
IP: CPS1
IP: CPS1
IB: CPS1
IB: CPS1
IB: CPS1
IB: CPS1
Input
IB: OGT
Input
IB: Myc
IB: GFP
IB: b-actin
Raw blot for Figure 3C
pFLAG-OGA
HeLa
-
IgG
+ (tet)
IB: RL-2
IP: CPS1
IB: CPS1
IB: OGA
FLAG-OGA
endo. OGA
Input
IB: CPS1
IB: b-actin

## Slide 7
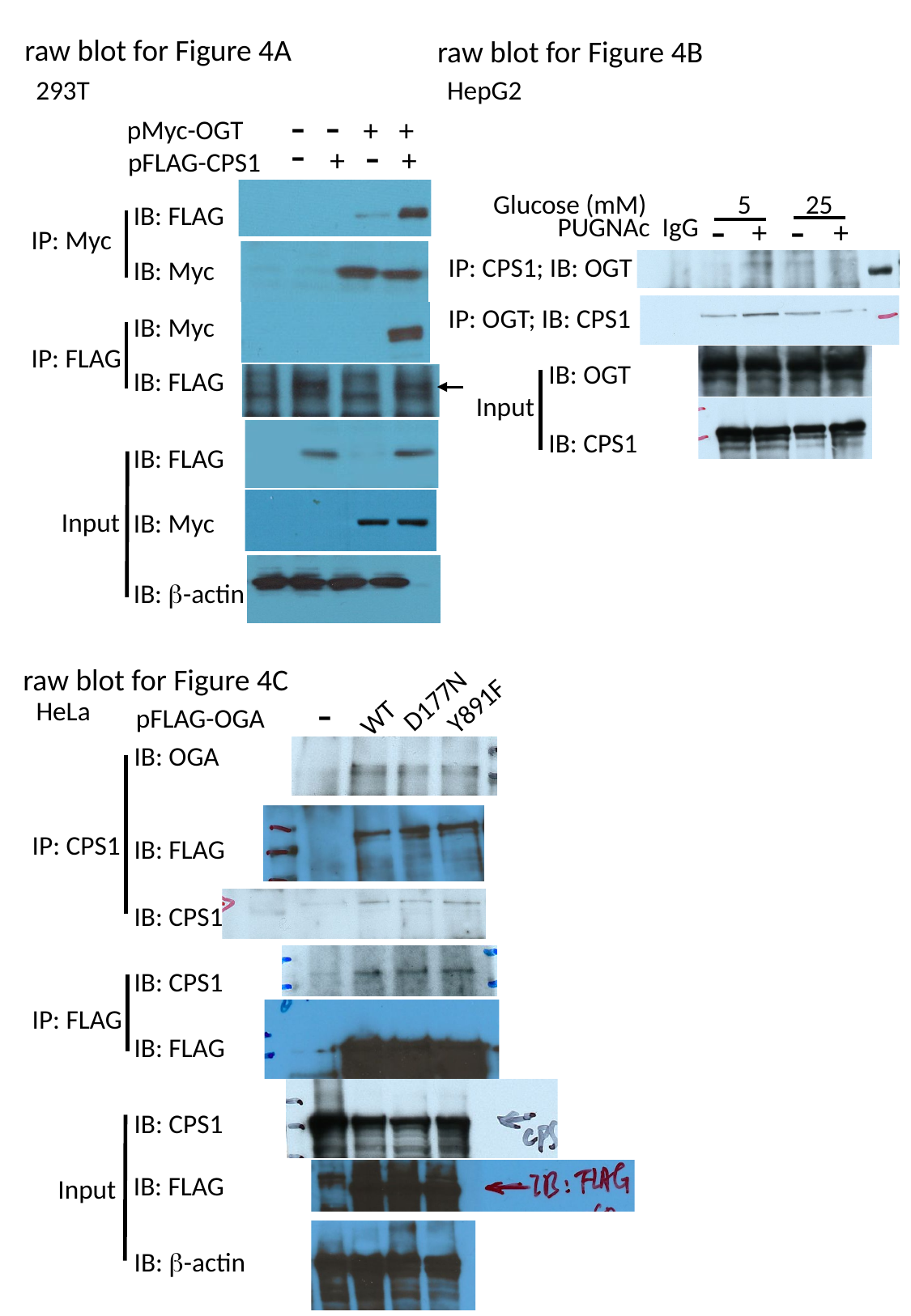

raw blot for Figure 4A
raw blot for Figure 4B
293T
HepG2
- -
+ +
pMyc-OGT
-
-
+
+
pFLAG-CPS1
Glucose (mM) 5 25
IB: FLAG
-
-
PUGNAc IgG
+
+
IP: Myc
IP: CPS1; IB: OGT
IB: Myc
IP: OGT; IB: CPS1
IB: Myc
IP: FLAG
IB: OGT
IB: FLAG
Input
IB: CPS1
IB: FLAG
Input
IB: Myc
IB: b-actin
raw blot for Figure 4C
D177N
-
Y891F
HeLa
WT
pFLAG-OGA
IB: OGA
IP: CPS1
IB: FLAG
IB: CPS1
IB: CPS1
IP: FLAG
IB: FLAG
IB: CPS1
IB: FLAG
Input
IB: b-actin

## Slide 8
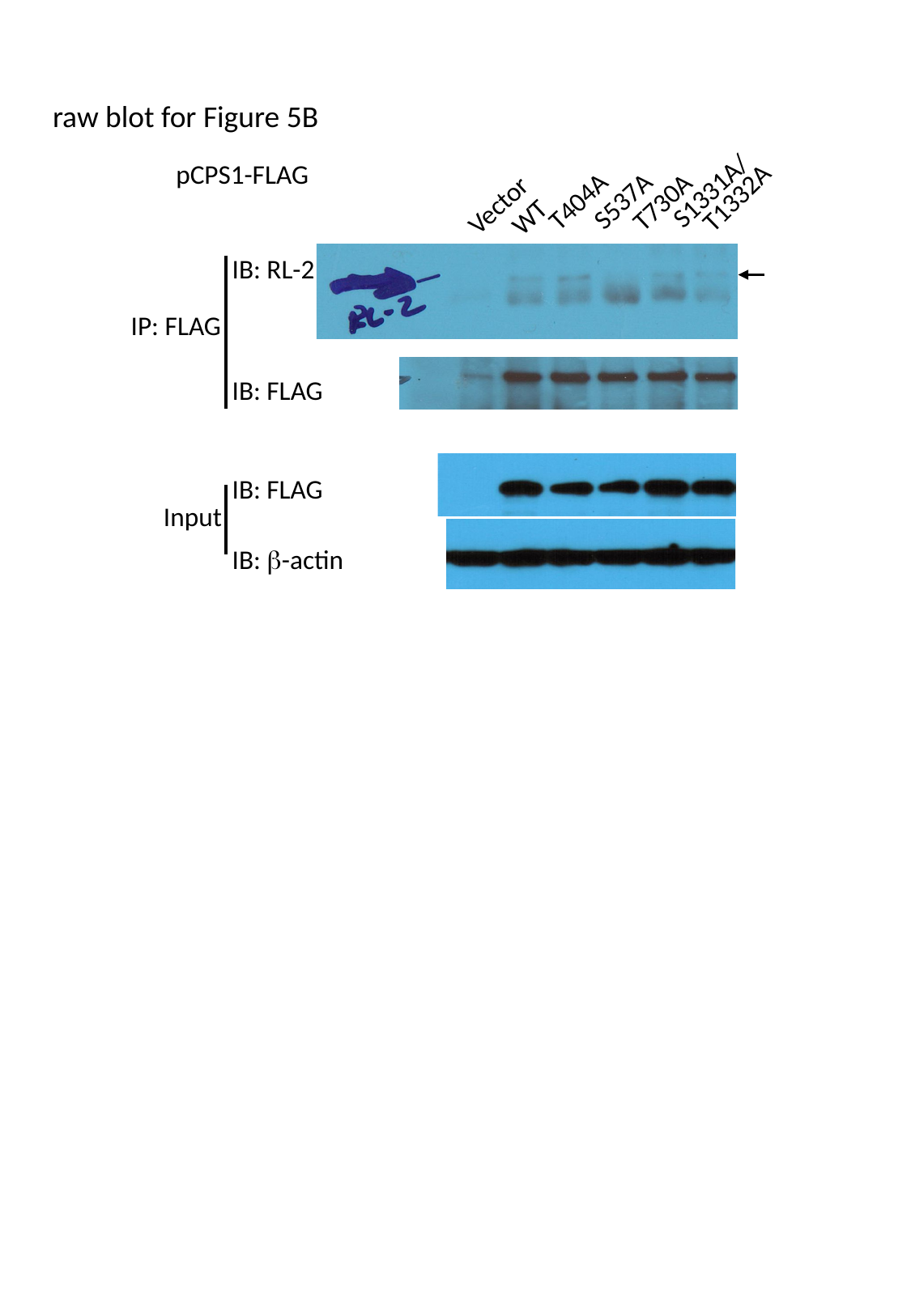

raw blot for Figure 5B
pCPS1-FLAG
S1331A/
T1332A
S537A
T404A
T730A
Vector
WT
IB: RL-2
IP: FLAG
IB: FLAG
IB: FLAG
Input
IB: b-actin

## Slide 9
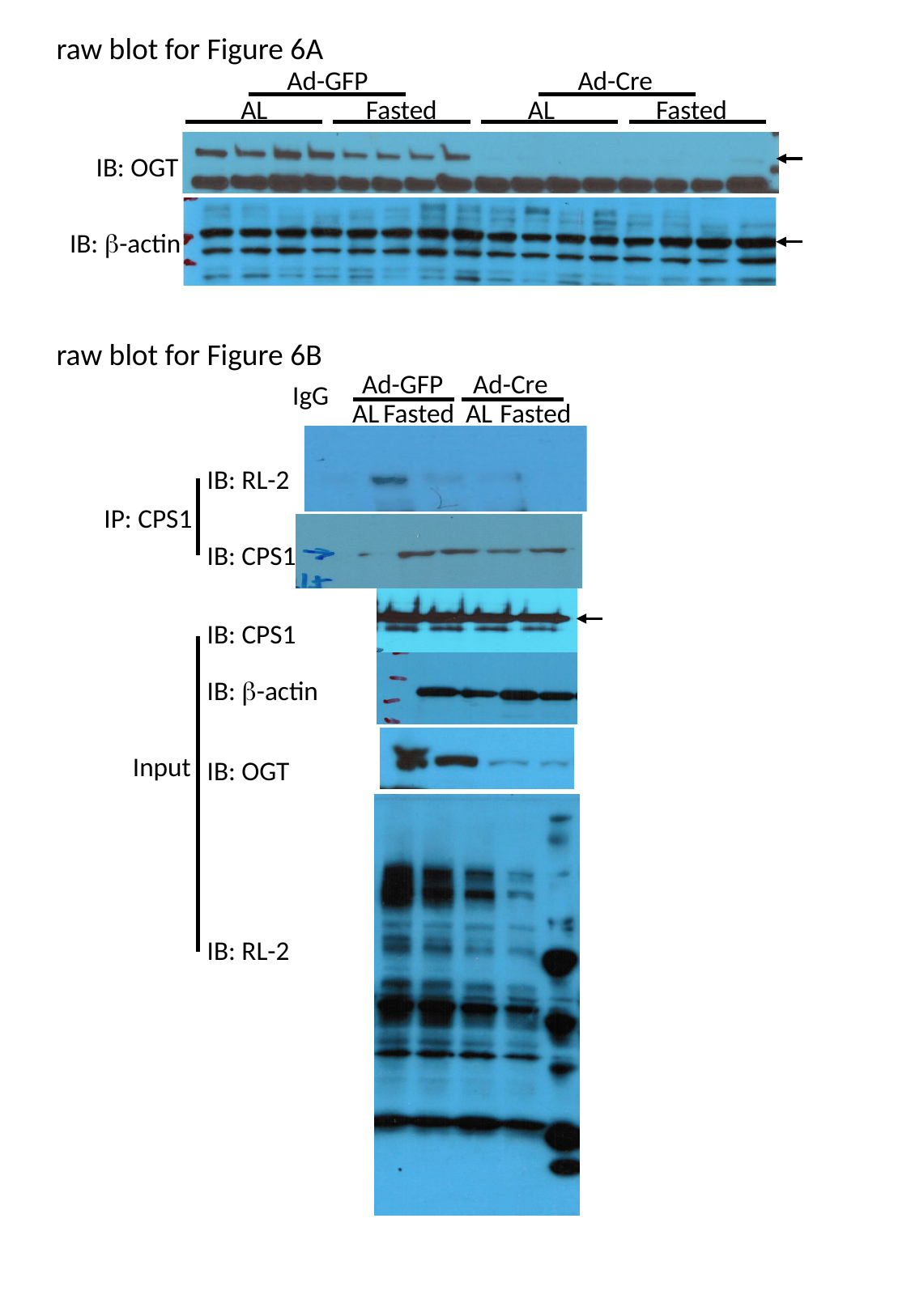

raw blot for Figure 6A
Ad-GFP
Ad-Cre
AL
Fasted
AL
Fasted
IB: OGT
IB: b-actin
raw blot for Figure 6B
Ad-GFP
Ad-Cre
IgG
AL
Fasted
AL
Fasted
IB: RL-2
IP: CPS1
IB: CPS1
IB: CPS1
IB: b-actin
Input
IB: OGT
IB: RL-2
